# Multimodal Analysis Reveals Immune Suppression Associated With Hepatocellular Carcinoma Related to RBM27 and Constructs a Prognostic Model

**DOI:** 10.1155/humu/4343678

**Published:** 2026-03-23

**Authors:** Wenjuan Gao, Dongwei Cong, Xiuqi Chen, Xiaofei Wang, Tilong Duan, Xiaochuan Zhao, Dandan Wang, Wei Liu, Chaoqun Huang

**Affiliations:** ^1^ Department of Gastroenterology, The 962th Hospital of the PLA Joint Logistic Support Force, Harbin, China; ^2^ Department of Infectious Diseases, The 962th Hospital of the PLA Joint Logistic Support Force, Harbin, China; ^3^ Department of Clinical Medicine, Heilongjiang Nursing College, Harbin, China

**Keywords:** genetic alteration, hepatocellular carcinoma, immune suppression, multiomics, oxidative phosphorylation, prognostic biomarker, RBM27

## Abstract

**Background:**

Hepatocellular carcinoma (HCC) exhibits aggressive progression and therapy resistance due to immunosuppressive microenvironments. RNA‐binding motif protein 27 (RBM27) is implicated in RNA processing, yet its role in HCC immune evasion remains uncharacterized.

**Methods:**

Multiomics analyses (TCGA/GEO, single‐cell/spatial transcriptomics) were integrated with functional validation (in vitro/vivo). RBM27 expression was assessed in HCC tissues/cell lines. Knockdown models (lentiviral shRNA) evaluated impacts on proliferation, migration, invasion (Transwell/wound healing), and tumor growth (xenografts). Immune profiling (ssGSEA/TIP), metabolic pathways (GSEA/KEGG), and prognostic modeling (Cox/nomogram) were performed. Spatial transcriptomics mapped immune niche alterations.

**Results:**

Compared with adjacent nontumor tissues, the expression level of RBM27 was markedly overexpressed in HCC tissues. Genomic analysis revealed that this upregulation is driven by copy number variations (CNVs), particularly gene amplification, alongside specific mutational patterns. This abnormal upregulation was closely correlated with advanced clinical stages of the disease, elevated AFP, and poor survival (OS/DSS/PFI; *p* < 0.05). RBM27 knockdown suppressed HCC proliferation, migration, invasion, and xenograft growth. Mechanistically, RBM27 activated oxidative phosphorylation, driving immunosuppression via CD8+ T cell and NK cell depletion, Treg/Th2 enrichment, and impaired cancer‐immunity cycle steps. Spatial analysis confirmed RBM27^+^ malignant niches with lymphoid exclusion. A prognostic nomogram (C − index = 0.688) incorporating RBM27 predicted 1‐/3‐/5‐year survival.

**Conclusion:**

Driven by genetic alterations including gene amplification, RBM27 promotes HCC progression by remodeling an immunosuppressive microenvironment via OXPHOS activation, acting as a biomarker for diagnosis and prognosis along with a potential therapeutic target, it could assist in combating immune escape.

## 1. Introduction

Hepatocellular carcinoma (HCC) ranks as the fourth leading cause of cancer‐associated mortality globally. Its high incidence and aggressiveness give it a significant clinical impact [1]. HCC exhibits a high postoperative recurrence rate, reaching 70% within 5 years, which severely compromises patient outcomes. Notably, HCC associated with hepatitis B virus (HBV) infection tends to present with aggressive features, including extensive tumor involvement, vascular infiltration, and rapid spread within the liver [2]. Patients with HCC still have an unfavorable prognostic outcome, despite progress in systemic treatment strategies, mainly due to the tumor′s strong invasive and metastatic capabilities and refractoriness to therapy. Consequently, the discovery of innovative biomarkers and molecular targets for both detection and treatment is crucial to enhance clinical management of patients with HCC.

RNA‐binding proteins (RBPs) play pivotal roles in posttranscriptional regulation, and their dysregulation is increasingly recognized as a key driver of cancer progression, among RBPs, the RBM family has emerged as critical regulators in multiple cancers, yet their specific roles in HCC metabolism and immune microenvironment remodeling remain incompletely defined [3]. Through preliminary bioinformatics screening of TCGA/GEO datasets, we identified RBM27 as a top candidate: It was significantly upregulated in HCC tissues relative to normal liver tissues, and its expression correlated with immune‐related pathways and oxidative phosphorylation (OXPHOS)—a metabolic pathway crucial for HCC cell survival and immunosuppression. Given the lack of studies investigating RBM27′s function in HCC, we aimed to systematically explore its oncogenic role, molecular mechanisms, and clinical relevance, with a focus on its potential involvement in immune microenvironment remodeling via metabolic regulation.

RBP encoded by RBM27 (RNA‐binding motif [RBM] protein 27) has been recognized as a potential autism spectrum disorder‐associated gene through research conducted by the Simons Foundation Autism Research Initiative (SFARI) [4]. Within human cell cultures, both RBM27 and its evolutionary counterpart RBM26 operate as components of the PAXT complex, serving as a recruitment platform for the nuclear RNA degradation machinery [5]. Similarly, in *Schizosaccharomyces pombe*, the RBM27 ortholog Rmn1 acts as an adaptor for the nuclear RNA exosome [6]. The *Drosophila* homolog Swm, corresponding to mammalian RBM26/27, participates in RNA regulatory processes and supports the homeostasis of intestinal progenitor cells in adults [7].

RBPs serve essential functions in modulating gene expression after transcription, influencing mRNA maturation, intracellular trafficking, subcellular distribution, and protein synthesis. These regulatory factors are classified into distinct groups according to their biological activities, such as Hu‐antigen R (HuR), heterogeneous nuclear ribonucleoproteins (hnRNPs), serine/arginine‐rich splicing regulators (SRSFs), and proteins containing RBMs [8]. Members of the RBM family demonstrate oncogenic associations across multiple malignancies, particularly hepatocellular, gastric, pulmonary, and mammary carcinomas. Their expression patterns correlate with clinical progression markers. Notably, Yong et al. observed reduced RBM4 levels in gastric tumors versus normal tissue, linking this suppression to aggressive features including undifferentiated histology, metastatic spread, and advanced TNM staging [9]. Similarly, Gao et al. demonstrated that RBM3 overexpression in colorectal cancer, gastric cancer, and melanoma correlates with improved prognosis [10]. Additionally, proteomic studies reveal RBM15 as a key WTAP interactor. As WTAP binds METTL3, the core m6A methyltransferase, RBM15 emerges as an essential member of this methylation complex. Patil et al. established its functional role in mediating m6A RNA modifications [10]. In investigations of RNA methylation regulatory factors in gastric cancer and papillary thyroid carcinoma (PTC), a marked positive association has been demonstrated between RBM15 expression levels and favorable prognostic outcomes [11, 12]. However, in lung adenocarcinoma (LUAD), high RBM15 expression is associated with poor prognosis [13, 14].

Despite the well‐established roles of RBM family proteins in cancer, the specific function of RBM27 in HCC remains unexplored. In this study, we utilized multiple databases to investigate the oncogenic role of RBM27 in HCC. Our research results indicate that RBM27 acts as a potential therapeutic target and diagnostic biomarker for HCC, with its downstream pathways and associated metabolic alterations presenting viable avenues for targeted therapy development.

## 2. Methods and Material

### 2.1. Database Source and Processing

HCC datasets underwent systematic reanalysis focusing on quantitative assessment of RBM27 transcript abundance (measured in transcripts per million, TPM) derived from high‐throughput RNA sequencing [15–17]. Gene expression profiles and related clinical data of 374 patients with HCC and 50 normal liver samples were collected from TCGA (https://portal.gdc.cancer.gov/) (Tables S1-S2). RNA‐seq data (TPM) were obtained together with information on the pertinent clinical aspects of the disease for patients with HCC. UALCAN, an interactive web portal comprising TCGA Level 3 RNA‐seq and clinical data from 33 cancer types, was used to analyze TCGA gene expression data in more depth. The UALCAN database (http://ualcan.path.uab.edu/index.html) was used to compare RBM27 transcription levels between HCC and normal liver samples as well as between diverse subtypes and substages. The datasets GSE76297, GSE39791, GSE121248, GSE57957, GSE84005, and GSE84598 were obtained from the GEO database (https://www.ncbi.nlm.nih.gov/geo) [18, 19].

### 2.2. Statistical Analysis

Statistical analyses were conducted using R software (v4.2.1), with the ggplot2 package (v3.4.4) applied for data visualization. For parametric and nonparametric comparisons, Student′s *t* tests, Kruskal–Wallis tests, or Fisher′s exact tests were utilized as appropriate. Survival differences were assessed through Kaplan–Meier estimation combined with log‐rank testing. Continuous data are presented as mean ± standard deviation (SD). The Cox proportional hazards regression model was used to assess the correlations between RBM27 expression levels and clinical outcomes, with two‐tailed *p* < 0.05 set as the threshold for statistical significance. Validation experiments were carried out with GraphPad Prism (v10.0) for independent verification of the results.

### 2.3. Additional Methods

Details of all procedures are presented in the Supporting Information.

## 3. Results

### 3.1. RBM27 Is Upregulated in HCC

To investigate RBM27 expression across various cancer types, we analyzed data from the TIMER database, which included 33 malignancies. Demonstrated findings indicated that RBM27 expression levels were markedly elevated in various cancer types, including cholangiocarcinoma (CHOL), esophageal carcinoma (ESCA), head and neck squamous cell carcinoma (HNSC), kidney renal clear cell carcinoma (KIRC), LUAD, liver hepatocellular carcinoma (LIHC), and stomach adenocarcinoma (STAD) (*p* < 0.05). Conversely, RBM27 expression was notably reduced in thyroid carcinoma (THCA) and uterine corpus endometrial carcinoma (UCEC) (Figure 1a). These findings were corroborated by TCGA data (Figure S1a). Additional investigation into RBM27 expression levels across 23 cancer types, focusing on paired tumor and adjacent normal tissues, has validated its marked overexpression in tumor tissues (Figure S1b). In HCC specifically, RBM27 expression was elevated in datasets from the UALCAN database and GEO datasets GSE76297 and GSE39791 (*p* < 0.001) (Figure 1b), as well as in additional datasets GSE121248, GSE57957, GSE84005, and GSE84598 (Figure S1c-f). Next, we extracted protein and mRNA samples from 20 pairs of HCC and adjacent normal tissues. RT‐qPCR confirmed that RBM27 mRNA expression was significantly higher in HCC tissues (*p* < 0.001) (Figure 1e), whereas Western blot analysis further verified its increased protein expression (Figure 1f). Furthermore, at both the mRNA and protein levels, the expression of RBM27 was elevated in several HCC cell lines (PLC/PRF/5, HCCLM3, MHCC97‐H, Huh7, and HepG2) compared with normal human hepatocytes (Wrl‐68) (Figure 1g,h). Immunohistochemical (IHC) staining further confirmed RBM27 upregulation in HCC tissues (Figure 1i). To assess the diagnostic potential of RBM27, we performed receiver operating characteristic (ROC) curve analysis. The area under the curve (AUC) for RBM27 was 0.804, indicating strong diagnostic value for distinguishing HCC from normal tissues (Figure 1j).

Figure 1The expression levels of RBM27 in HCC and cell lines were verified by TCGA and GEO databases and in vitro experiments. (a) Comparison of RBM27 expression levels in different cancer tissues and normal tissues. (b–d) TCGA and GEO databases were used to analyze the expression of RBM27 in HCC tissues. (e) RT‐qPCR and (f) Western blot were used to detect the mRNA and protein expression levels of RBM27 in HCC and adjacent tissues (*n* = 20). The mRNA and protein expression levels of RBM27 in WRL‐68, PLC/PRF/5,HCCLM3, MHCCH97‐H, Huh7, and HepG2 cell lines were detected by (g) RT‐qPCR and (h) Western blot, respectively. (i) RBM27 protein levels in normal liver and HCC were measured using IHC. (j) ROC curve was used to analyze the value of RBM27 in differentiating HCC tissues.  ^∗^ *p* < 0.05,  ^∗∗^ *p* < 0.01,  ^∗∗∗^ *p* < 0.001, NS, no significance.

**Figure:**
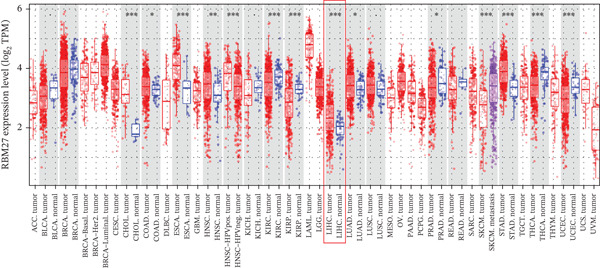
(a)

**Figure:**
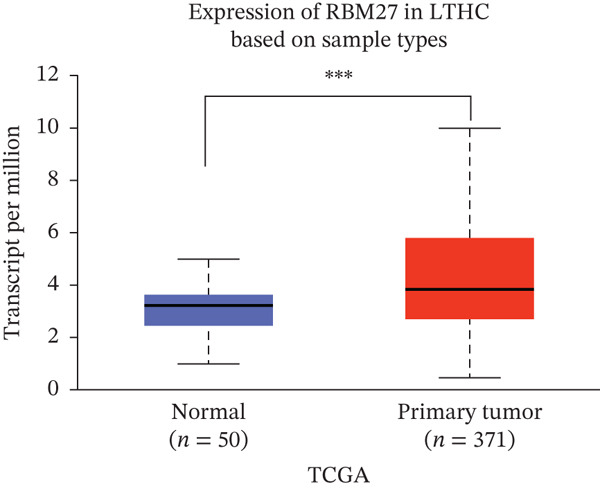
(b)

**Figure:**
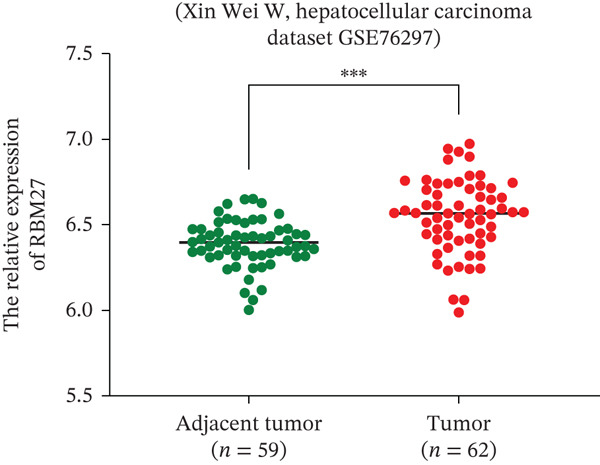
(c)

**Figure:**
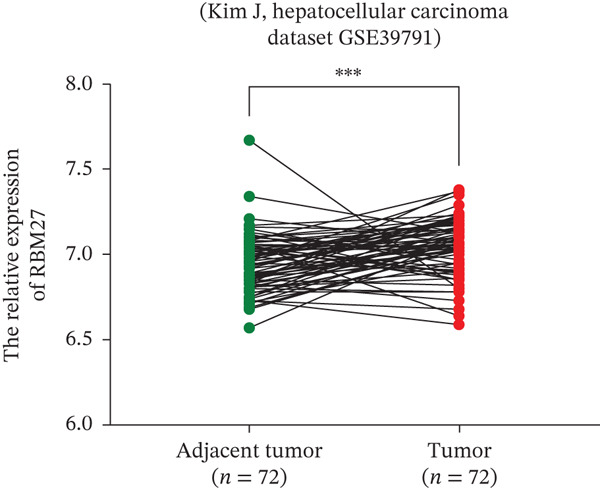
(d)

**Figure:**
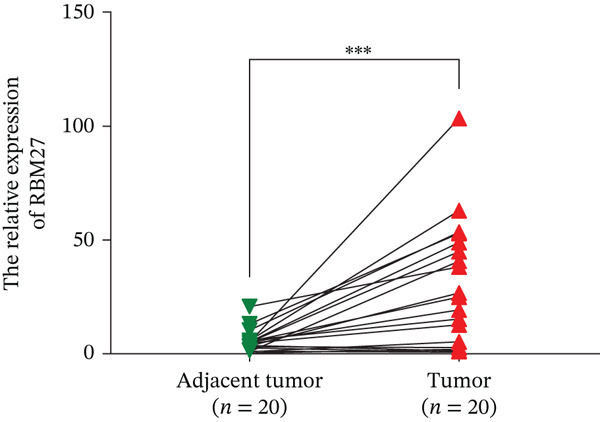
(e)

**Figure:**
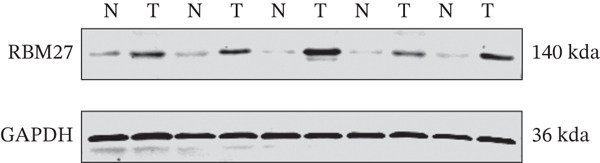
(f)

**Figure:**
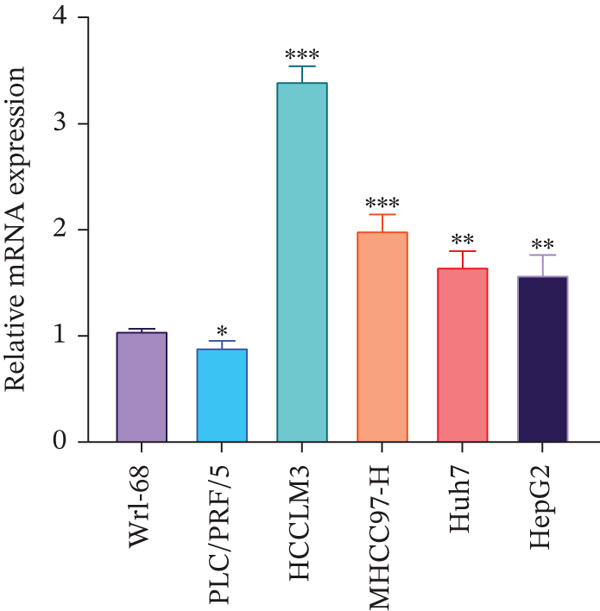
(g)

**Figure:**
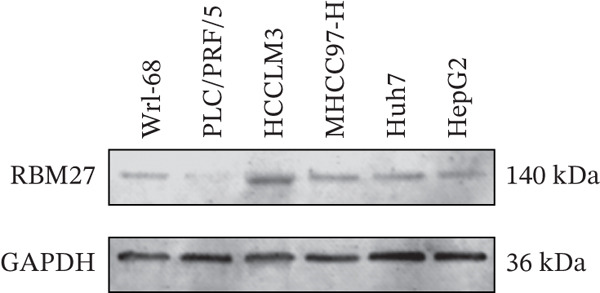
(h)

**Figure:**
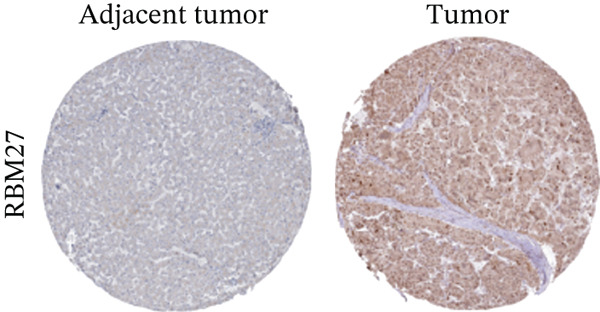
(i)

**Figure:**
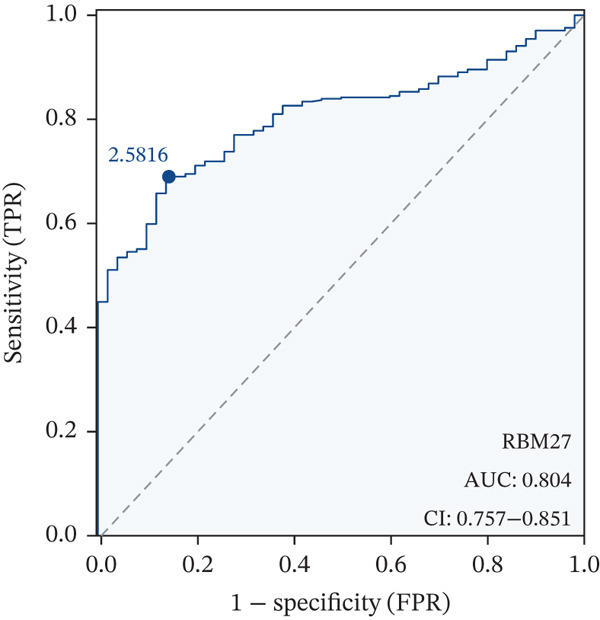
(j)

### 3.2. Prognostic Value of RBM27 in HCC

Kaplan–Meier survival analysis was conducted to evaluate the correlation between RBM27 expression levels and the prognostic outcomes of patients with HCC. In these patients, elevated RBM27 expression exhibited a significant association with unfavorable overall survival (OS), disease‐specific survival (DSS), and progression‐free interval (PFI) (Figures 2a, 2b, and 2c). A forest plot further confirmed that elevated RBM27 expression serves as a risk factor for OS (Figure 2d), DSS (Figure 2e), and PFI (Figure 2f). To establish a predictive model, a nomogram was constructed using RBM27 expression along with other independent clinicopathological factors. Each variable was assigned a score based on the nomogram scale, and the total score was used to estimate 1‐, 3‐, and 5‐year survival probabilities. The concordance index (C‐index) of the nomogram was 0.688 (95% CI: 0.659–0.716), indicating good predictive accuracy (Figure 2g). Calibration curves demonstrated a strong agreement between predicted and observed survival outcomes (Figure 2h), further validating the model′s reliability. Such outcomes were in line with the results of the univariate Cox regression analysis.

Figure 2Prognostic value of RBM27 in HCC. (a–c) Survival curves show comparison of OS, DSS, and PFI in patients with HCC with high versus low RBM27 expression. (d–f) Univariate survival analysis of OS, PFl, and DSS in different subgroups of patients stratified by TNM stage, pathological grade, tumor status, and RBM27 expression level. (g) A nomogram was constructed to estimate the 1‐, 3‐, and 5‐year OS probabilities. (h) Nomogram calibration plot used to determine 1‐, 3‐, and 5‐year OS probabilities. ^∗^ *p* < 0.05;  ^∗∗^ *p* < 0.01;  ^∗∗∗^ *p* < 0.001. NS, no significance.

**Figure:**
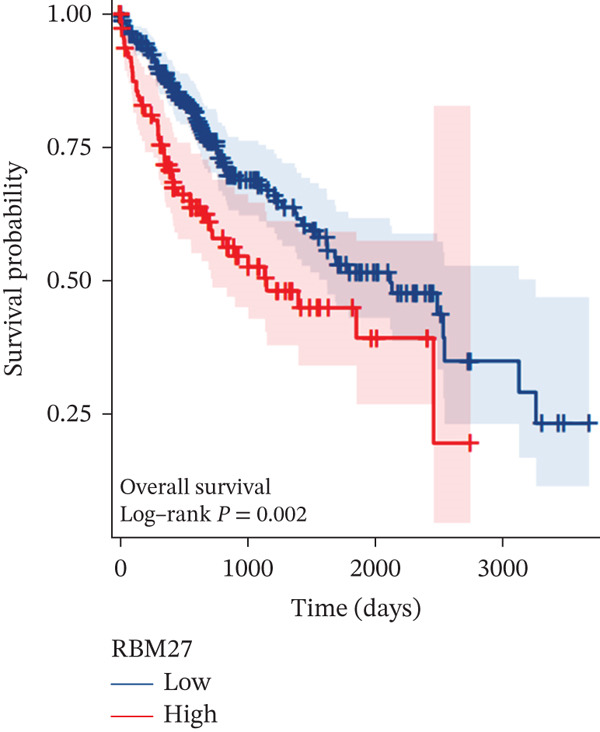
(a)

**Figure:**
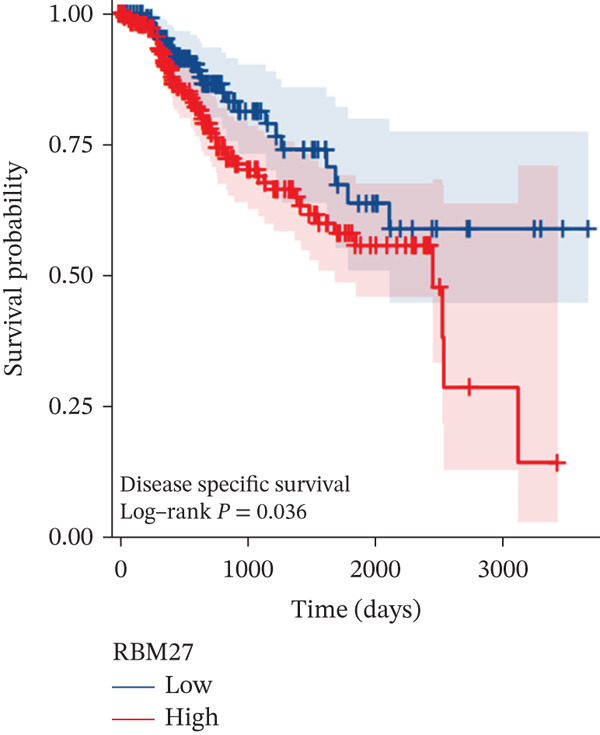
(b)

**Figure:**
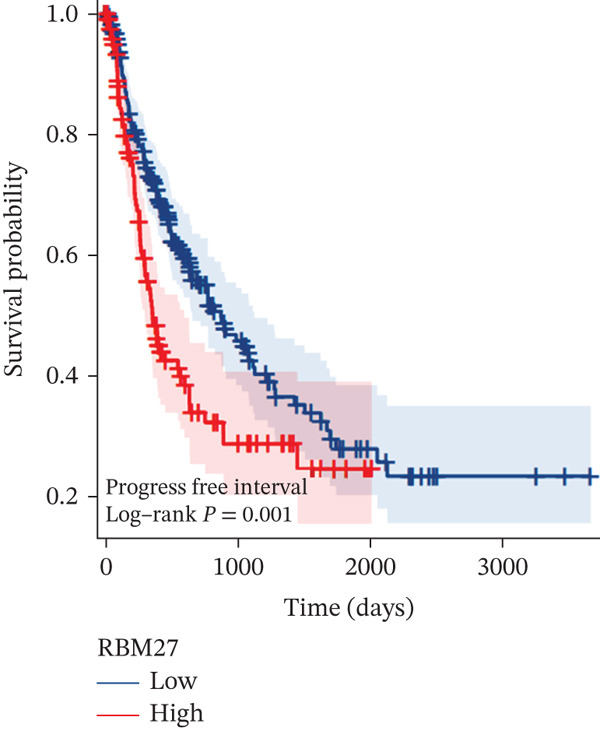
(c)

**Figure:**
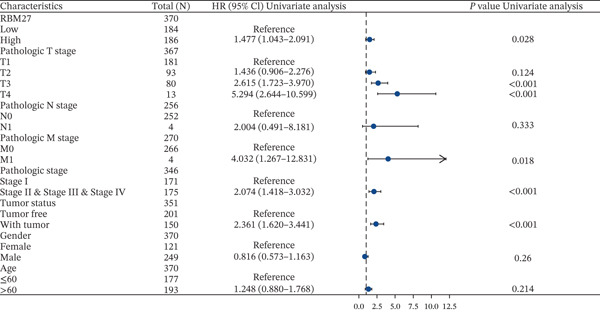
(d)

**Figure:**
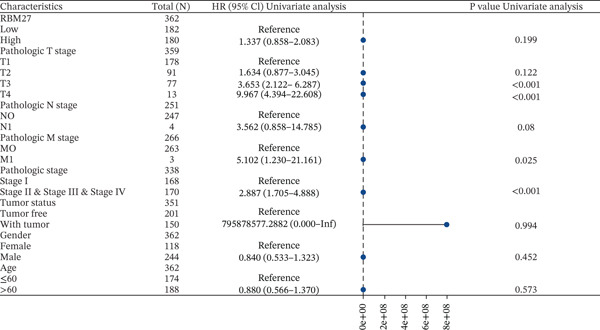
(e)

**Figure:**
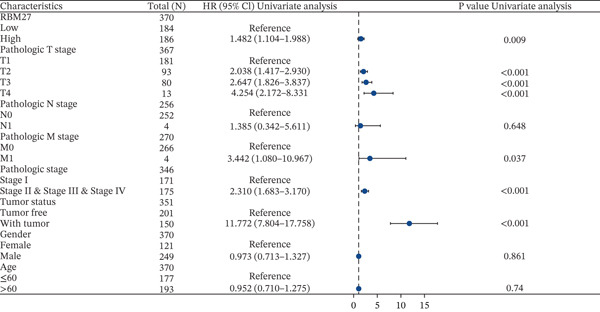
(f)

**Figure:**
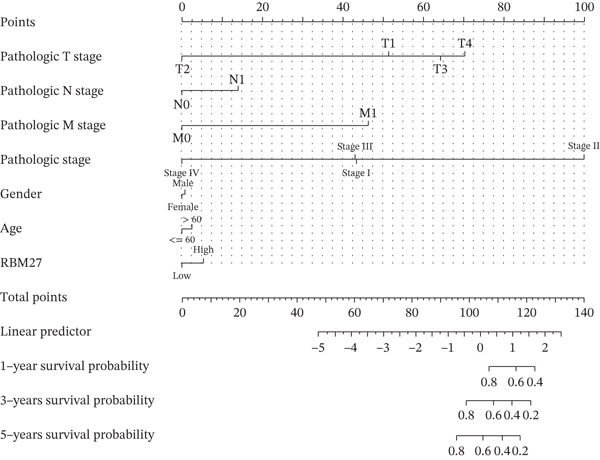
(g)

**Figure:**
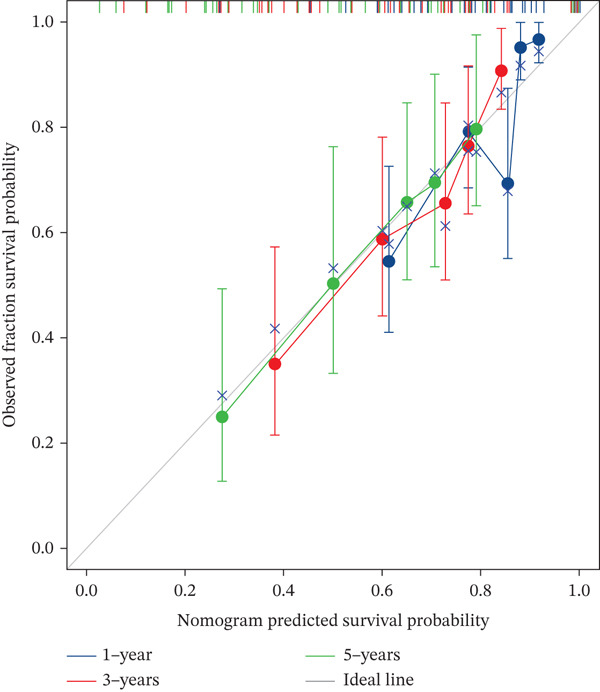
(h)

Logistic regression analysis revealed that elevated RBM27 expression in HCC was significantly associated with advanced pathological T stage (T3 and T4 vs. T1 and T2; OR = 1.611, *p* = 0.014), lower body weight (OR = 0.594 for weight > 70 kg vs. ≤ 70 kg, *p* = 0.017), increased alpha‐fetoprotein (AFP) levels (OR = 1.983 for AFP > 400 ng/mL vs. ≤ 400 ng/mL, *p* = 0.018), and tumor status (OR = 0.547 for patients with tumors vs. tumor‐free, *p* = 0.040) (Table 1).

**Table 1:** Logistic regression analysis revealed that elevated RBM27 expression in HCC.

Characteristics	Total (*N*)	OR (95% CI)	*p*value
Pathologic T stage (T1and T2 vs. T3 and T4)	371	0.547 (0.339–0.883)	0.014
Pathologic *N* stage (N0 vs. N1)	258	0.366 (0.038–3.569)	0.387
Pathologic *M* stage (M0 vs. M1)	272	0.338 (0.035–3.294)	0.351
Pathologic stage (Stage I and Stage II vs. Stage III and Stage IV)	350	0.512 (0.314–0.837)	0.008
Gender (male vs. female)	374	0.692 (0.448–1.070)	0.098
Tumor status (with tumor vs. tumor free)	355	1.556 (1.019–2.374)	0.040
Race (Black or African American and White vs. Asian)	362	0.946 (0.625–1.433)	0.794
Age (> 60 vs. ≤ 60)	373	0.797 (0.531–1.198)	0.275
Weight (> 70 vs. ≤ 70)	346	0.594 (0.388–0.910)	0.017
Height (≥ 170 vs. < 170)	341	0.720 (0.467–1.111)	0.138
BMI (>25 vs. ≤ 25)	337	0.719 (0.468–1.104)	0.132
AFP (ng/mL) (> 400 vs. ≤ 400)	280	1.983 (1.124–3.498)	0.018
Child–Pugh grade (A vs. B and C)	241	0.974 (0.404–2.348)	0.997
Albumin (g/dL) (≥ 3.5 vs. < 3.5)	300	1.344 (0.781–2.314)	0.286
Vascular invasion (Yes vs. No)	318	1.039 (0.655–1.650)	0.870

### 3.3. Correlation Between RBM27 Expression and Clinical Characteristics

To further explore the clinical significance of RBM27, we analyzed TCGA clinical data after excluding cases with incomplete information. A total of 374 patients with HCC were included, with a mean age of 61.5 years (range: 49.25–70.00 years) and a male‐to‐female ratio of 2:1. Stratification of patients based on RBM27 expression levels revealed significant associations with TNM stage, tumor status, gender, age, BMI, pathological stage, fibrosis risk score, vascular invasion, and AFP levels (Figures 3a, 3b, 3c, 3d, 3e, 3f, 3g, 3h, 3i, 3j, 3k, and 3l). Notably, RBM27 expression was significantly correlated with tumor status (*p* = 0.040) and body weight (*p* = 0.016). Furthermore, patients with AFP levels > 400 ng/mL exhibited significantly higher RBM27 expression (*p* = 0.017). These research results indicate that patients with HCC with elevated RBM27 expression levels are prone to present with more progressed clinical disease stages.

Figure 3Correlation of RBM27 expression with clinicopathological characteristics. (a) T stage. (b) N stage. (c) M stage. (d) Pathological stage. (e) Tumor status. (f) Gender. (g) Age. (h) BMI. (i) Weight. (j) Vascular invasion. (k) AFP level. (l) Fibrosis risk score (Ishak fibrosis score grouping: 0 = *no fibrosis*; 1–2 = *mild fibrosis*; 3–4 = *moderate fibrosis*; 5–6 = *cirrhosis*). ^∗^ *p* < 0.05,  ^∗∗^ *p* < 0.01,  ^∗∗∗^ *p* < 0.001.

**Figure:**
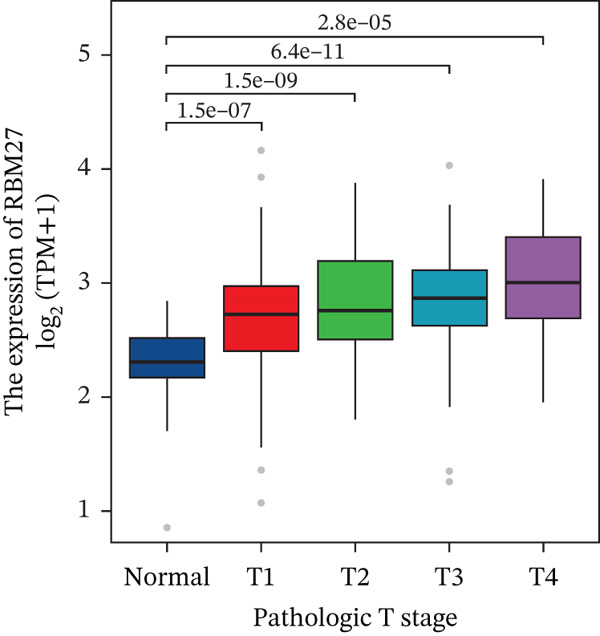
(a)

**Figure:**
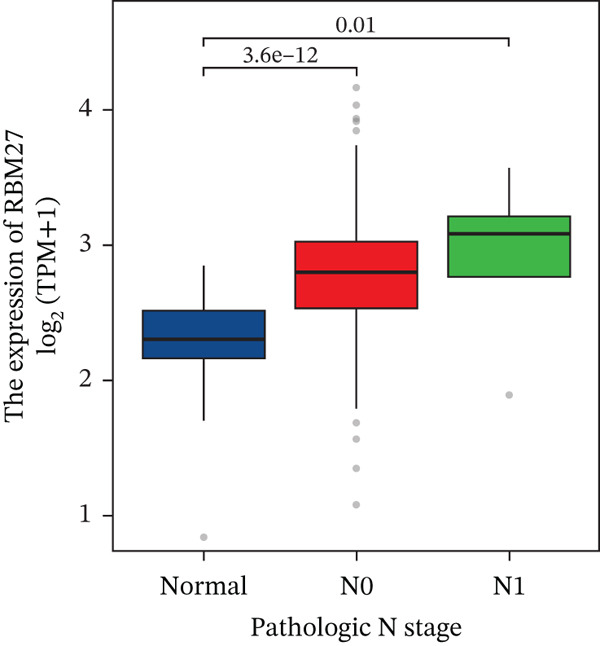
(b)

**Figure:**
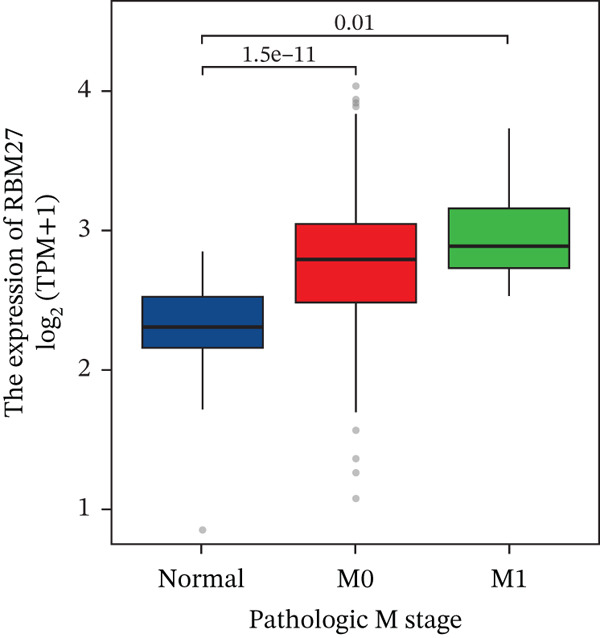
(c)

**Figure:**
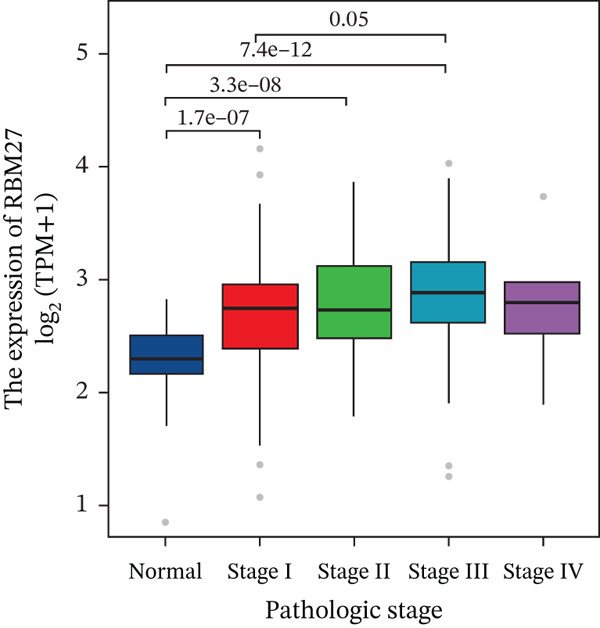
(d)

**Figure:**
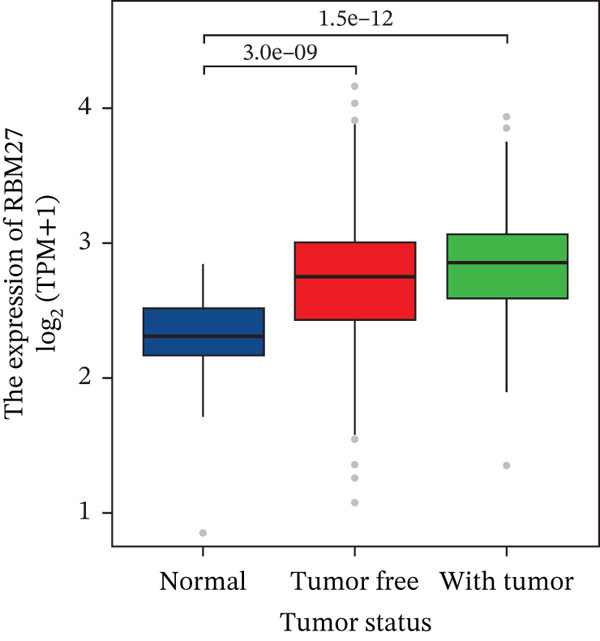
(e)

**Figure:**
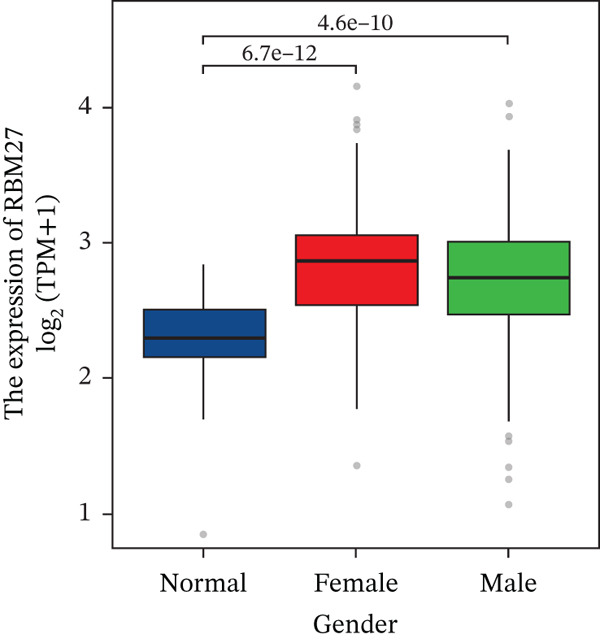
(f)

**Figure:**
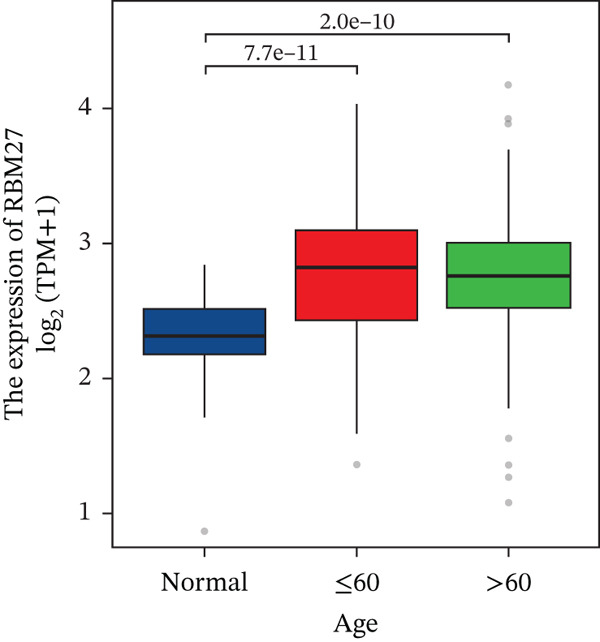
(g)

**Figure:**
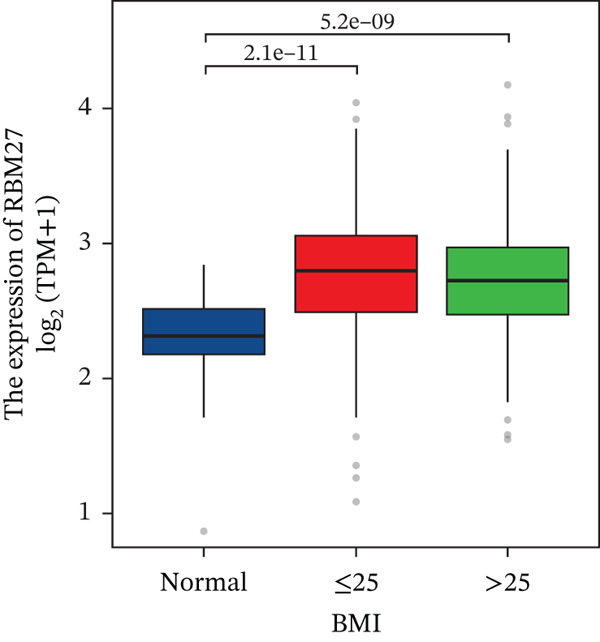
(h)

**Figure:**
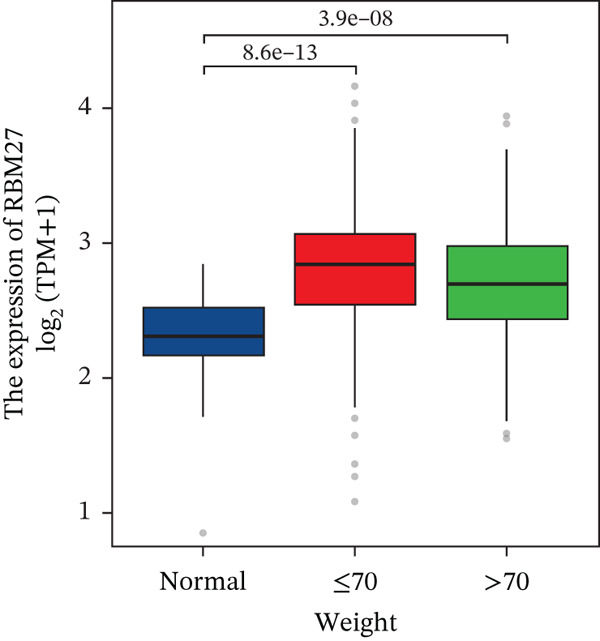
(i)

**Figure:**
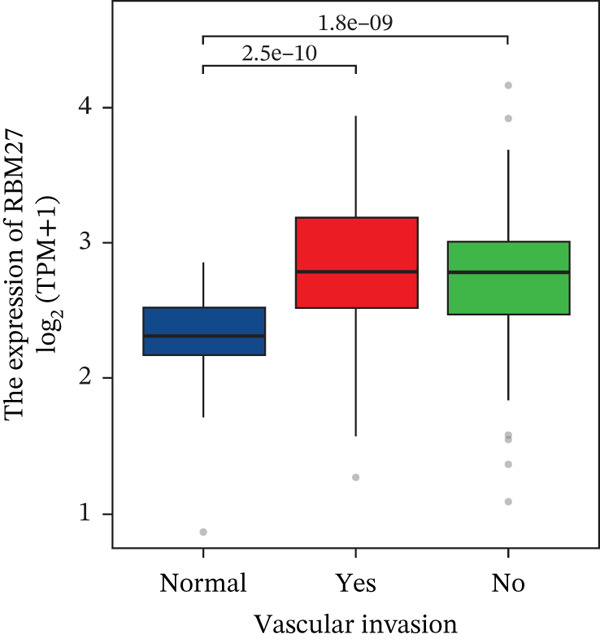
(g)

**Figure:**
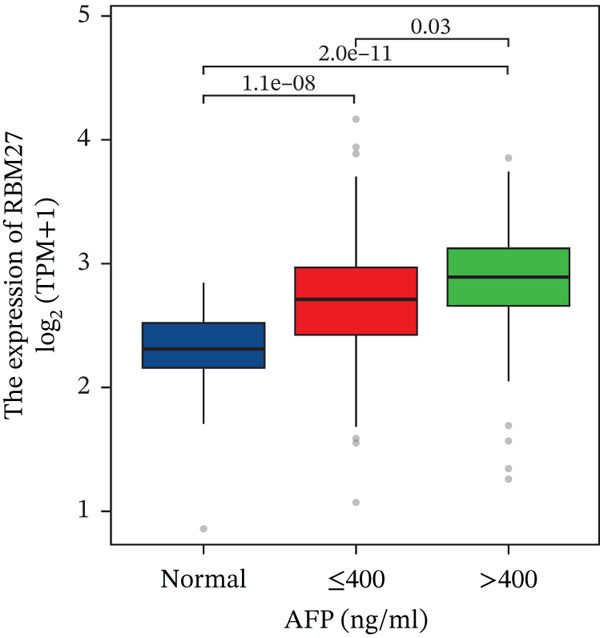
(k)

**Figure:**
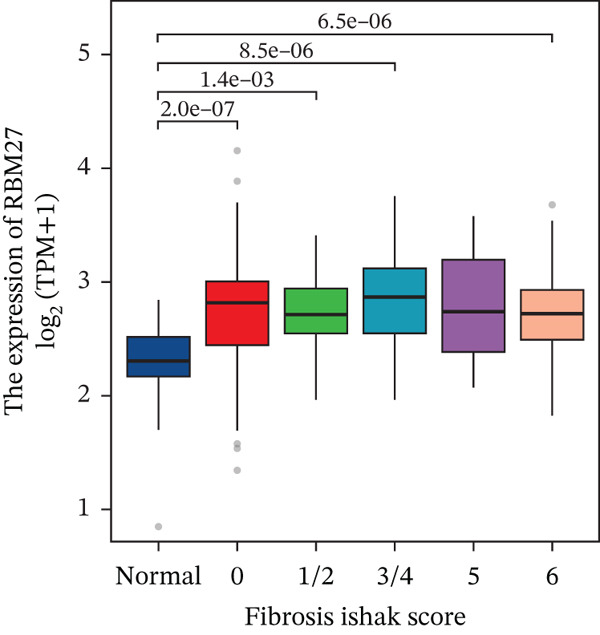
(l)

### 3.4. Genetic Alterations in RBM27 Across Cancer Types

Using cBioPortal, we analyzed RBM27 genetic alterations in a dataset comprising 10,967 cancer samples. Genomic variations in RBM27 were detected in 2.5% (279 cases) of patients (Figure 4a), with the highest mutation frequency observed in female patients with endometrial cancer (10.02%), and also in HCC (1.34%) (Figure 4b). A total of 232 mutation sites were identified, including 165 missense mutations, two in‐frame mutations, 54 truncating mutations, 10 splicing mutations, and one fusion mutation (Figure 4c). Copy number variation (CNV) analysis revealed that approximately 9% of samples exhibited gene deletions with expression levels below the diploid threshold. Conversely, gene amplification (gain and amplification events) was detected in nearly one‐third of samples (*p* < 0.001) (Figure 4d,e).

Figure 4RBM27 gene mutation frequency, type, and its relationship with expression level. (a) Frequency of RBM27 gene mutations in 10,967 samples. (b) Frequency of RBM27 gene mutations in various cancers. (c) Mutation types and proportions of RBM27. (d–e) Relationship between copy number and mRNA expression of RBM27 and gene mutations.

**Figure:**
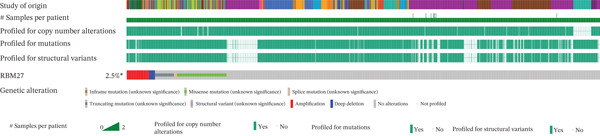
(a)

**Figure:**
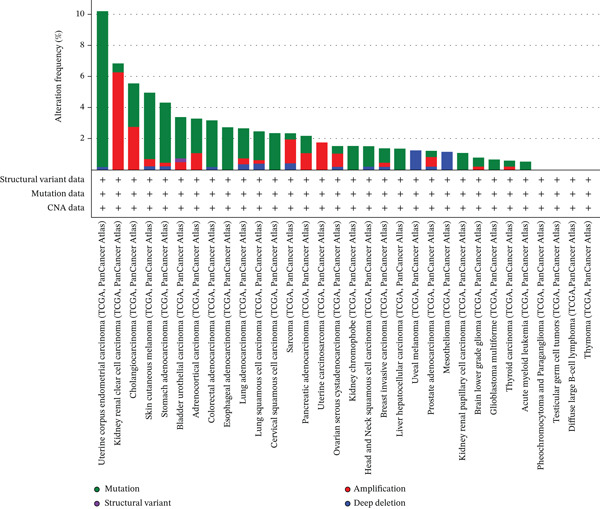
(b)

**Figure:**
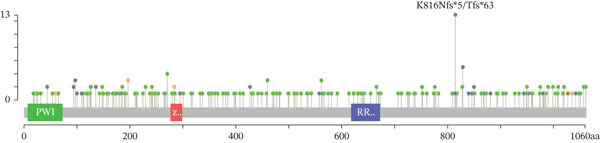
(c)

**Figure:**
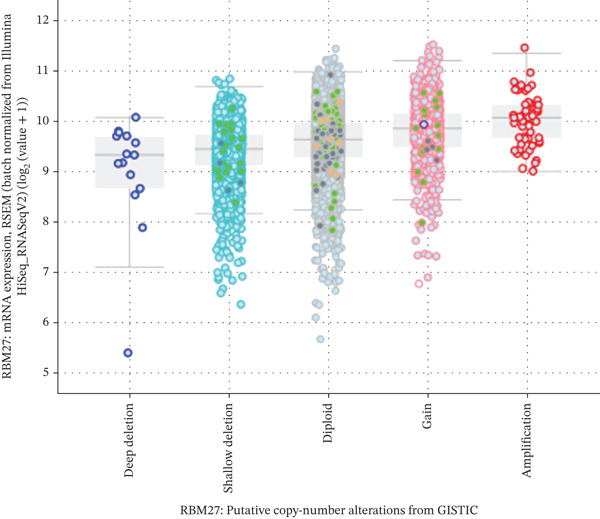
(d)

**Figure:**
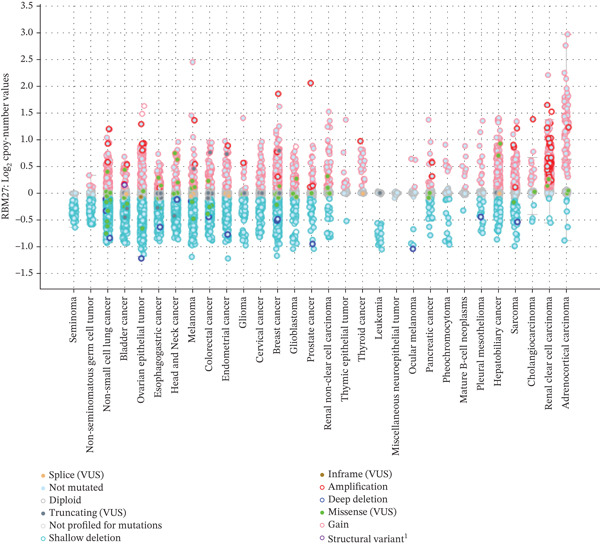
(e)

### 3.5. Protein–Protein Interaction (PPI) Network Analysis of RBM27

Using the STRING database, we identified nine key genes that exhibit functional interactions with RBM27. A PPI network was constructed using Cytoscape, illustrating these associations (Figure 5a,b). Next, we quantified the expression of these 10 candidate genes in TCGA in HCC specimens and found that among the identified interacting genes, expression was elevated in HCC tumor tissues, except for PABPN1L, RBMXL1 (Figure 5c). To delineate the molecular regulatory network associated with RBM27 in HCC, we analyzed the coexpression profiles of RBM27 with 10 functionally relevant target genes. Our data demonstrated that RBM27 mRNA transcript levels were markedly positively associated with 10 genes (*p* < 0.05) (Figures 5d, 5e, 5f, 5g, 5h, 5i, 5j, and 5k). GO enrichment analysis revealed that genes interacting with RBM27 are primarily involved in mRNA processing, RNA splicing, and mRNA metabolism regulation (Figure 5l). To further investigate RBM27 interactions, we utilized AlphaFold3 to predict its binding sites with ZC3H18, the gene with the highest correlation score (Figure 5m).

Figure 5PPI network enrichment analysis. (a) The PPI network was built based on PPI pairs identified by the STRING dataset. (b) String network protein interaction score. (c) Expression profiles of 10 genes functionally associated with RBM27 in HCC from TCGA. (d–k) Correlation heatmaps demonstrating coexpression patterns betweenRBM27 and its associated genes. (l) GO enrichment analysis was used to analyze the proteins that interacted with RBM27. (m) Protein docking sites were predicted by Alfhafold3 website.

**Figure:**
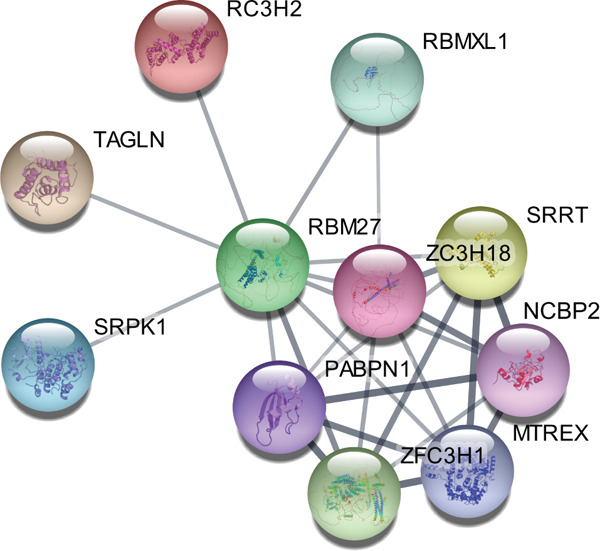
(a)

**Figure:**
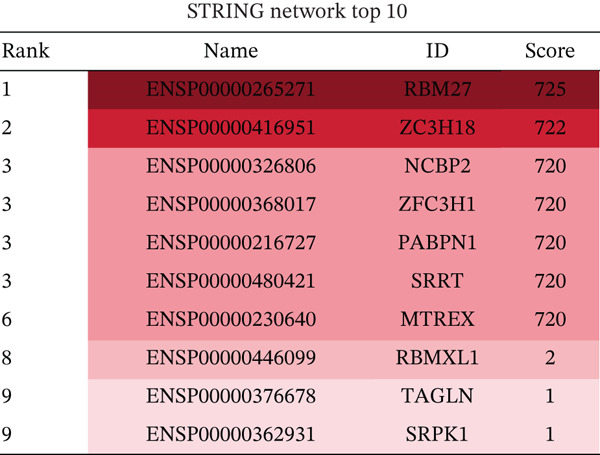
(b)

**Figure:**
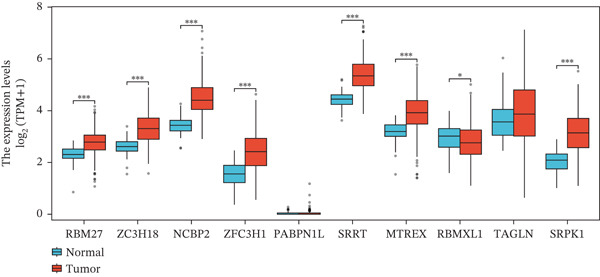
(c)

**Figure:**
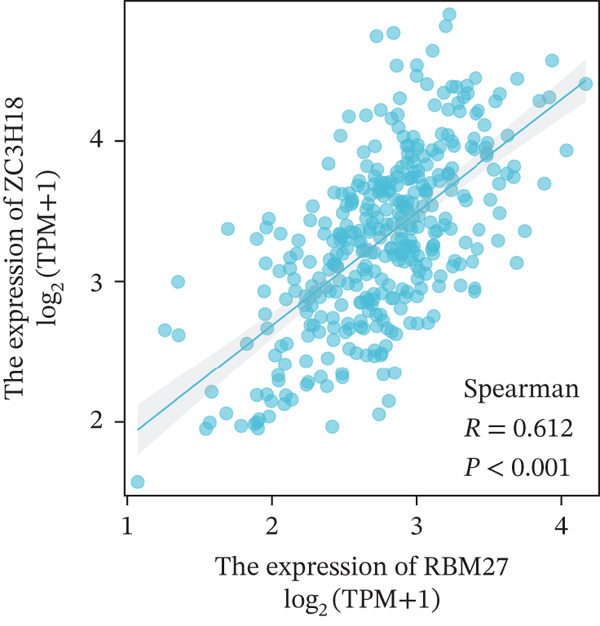
(d)

**Figure:**
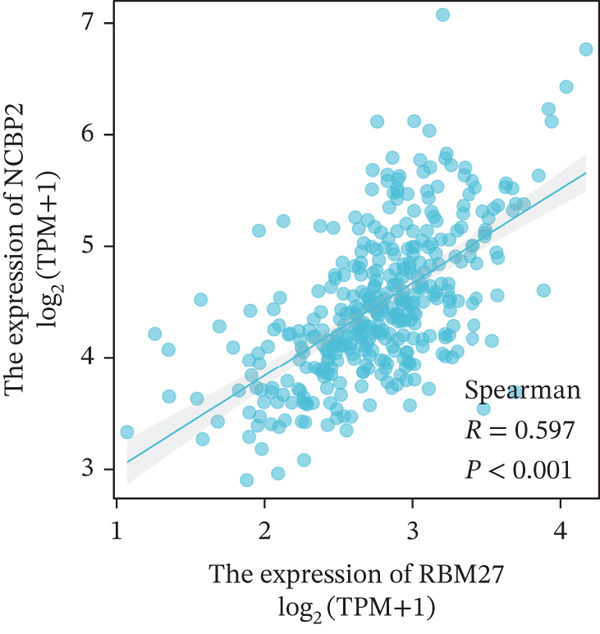
(e)

**Figure:**
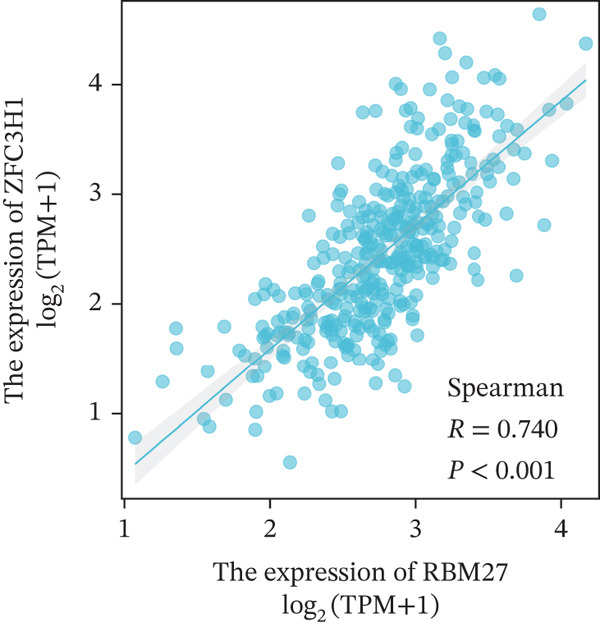
(f)

**Figure:**
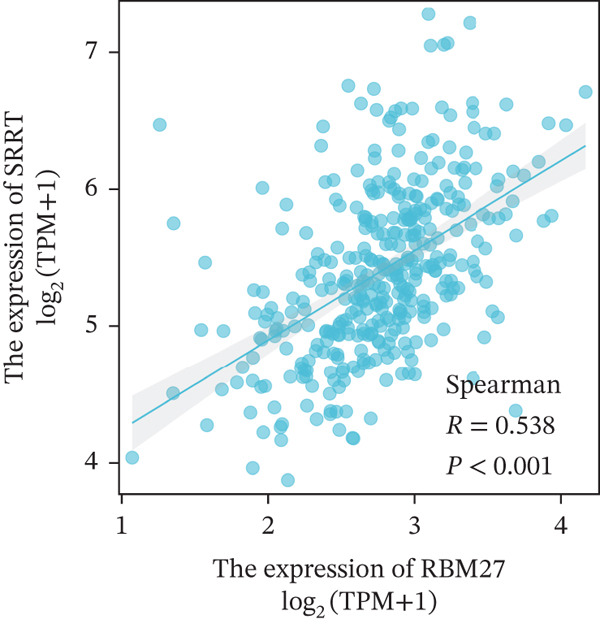
(g)

**Figure:**
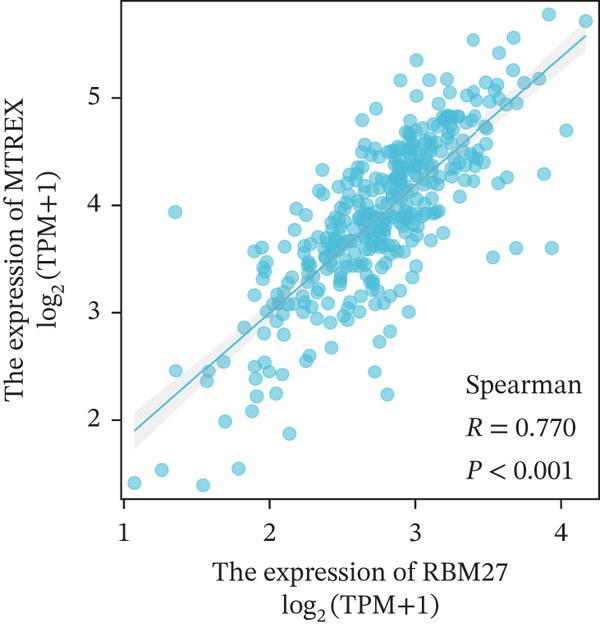
(h)

**Figure:**
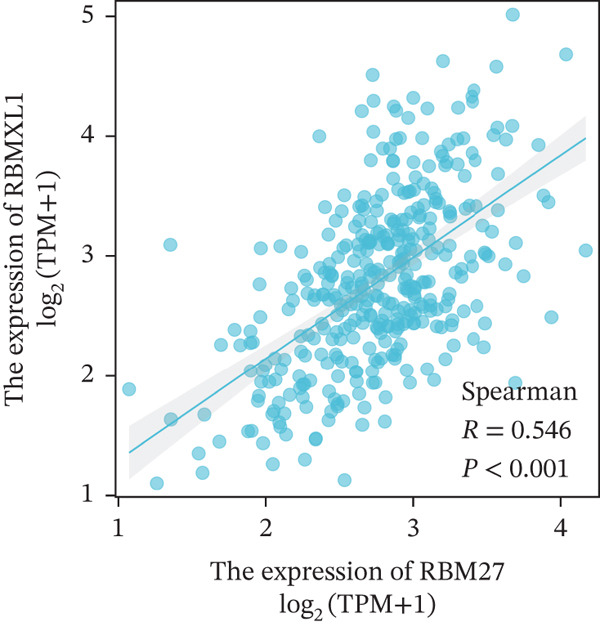
(i)

**Figure:**
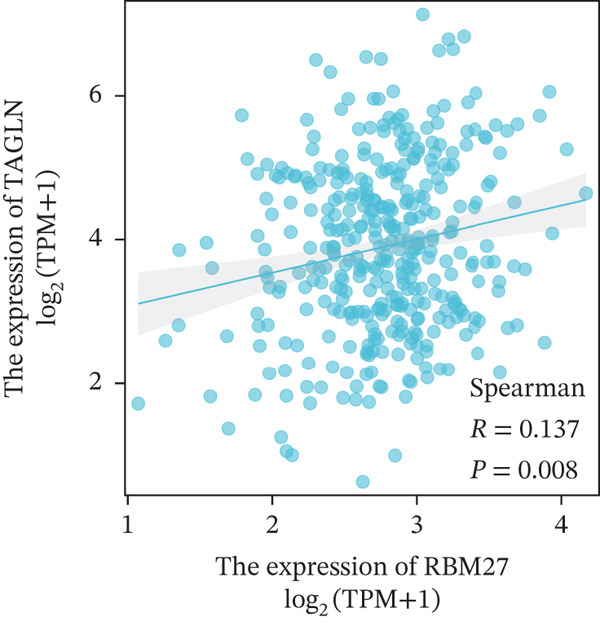
(j)

**Figure:**
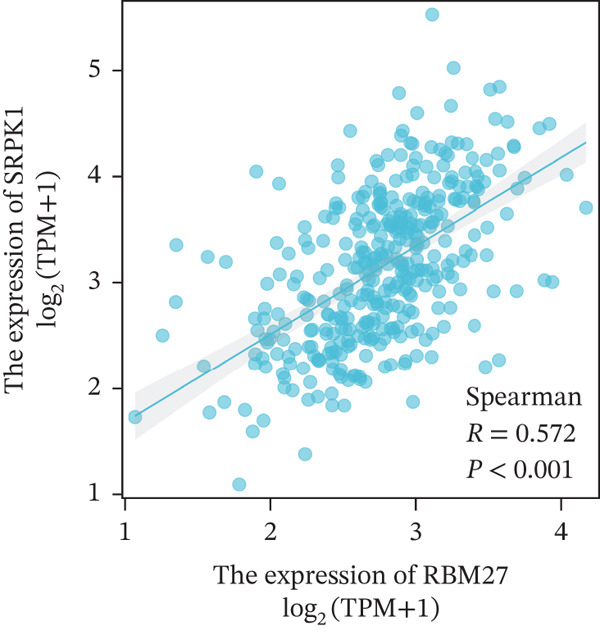
(k)

**Figure:**
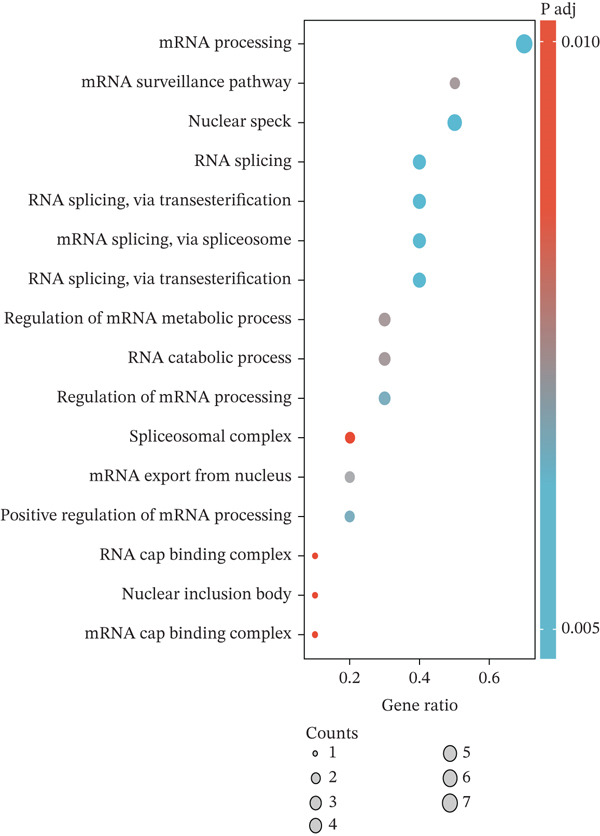
(l)

**Figure:**
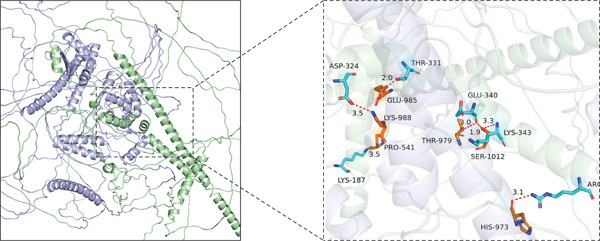
(m)

### 3.6. RBM27 Orchestrates Immunosuppressive Reprogramming to Drive Immune Evasion in HCC

Integrated analysis of immunomodulator dynamics in HCC revealed that RBM27‐high tumors exhibit coordinated alterations favoring immune evasion. These include elevated amplification of immunosuppressive checkpoints, epigenetic silencing of T‐cell costimulatory signals evidenced by strong negative expression‐methylation correlations, and increased deletion frequencies of proinflammatory chemokines. This multidimensional dysregulation in tumors with RBM27 abnormalities fosters an immunosuppressive tumor microenvironment (TME) that facilitates tumor immune escape (Figure 6a). Tumor immune phenotype (TIP) analysis across the cancer‐immunity cycle further demonstrates that elevated RBM27 expression exerts broad suppressive effects on key antitumor immune processes. It exhibits significant negative correlations with T cell priming/activation, immune cell trafficking to tumors, and effector cell infiltration into the TME. Moreover, RBM27 associates with impaired recruitment of cytotoxic CD8^+^ T cells and NK cells while concurrently exhibiting coordinated immunomodulator dysregulation that favors checkpoint activation and chemokine suppression (Figure 6b). Characterization of immune response and genomic state shows RBM27‐high tumors (Q1) display a distinct immunosuppressive phenotype, characterized by significantly reduced lymphocyte infiltration, impaired IFN‐*γ* response, and diminished leukocyte fraction relative to RBM27‐low tumors. These tumors concurrently exhibit lower stromal fraction and decreased intratumor heterogeneity, indicating a less immunogenic microenvironment. The concomitant reduction in B‐cell receptor diversity (BCR evenness) additionally suggests compromised adaptive immunity (Figure 6c).

Figure 6Multimodal immunomodulator dysregulation in RBM27‐high HCC. (a) Multimodal immunomodulator dysregulation in RBM27‐high HCC. (b) Suppressive impact of RBM27 on the cancer‐immunity cycle. (c) Immunosuppressive phenotype of RBM27‐high tumors. (d) Altered immune subtype distribution in RBM27‐high hepatocellular carcinoma. (C2 is IFN‐*γ*‐dominant, C3 is inflammatory, C4 is lymphocyte‐depleted [20]).

**Figure:**
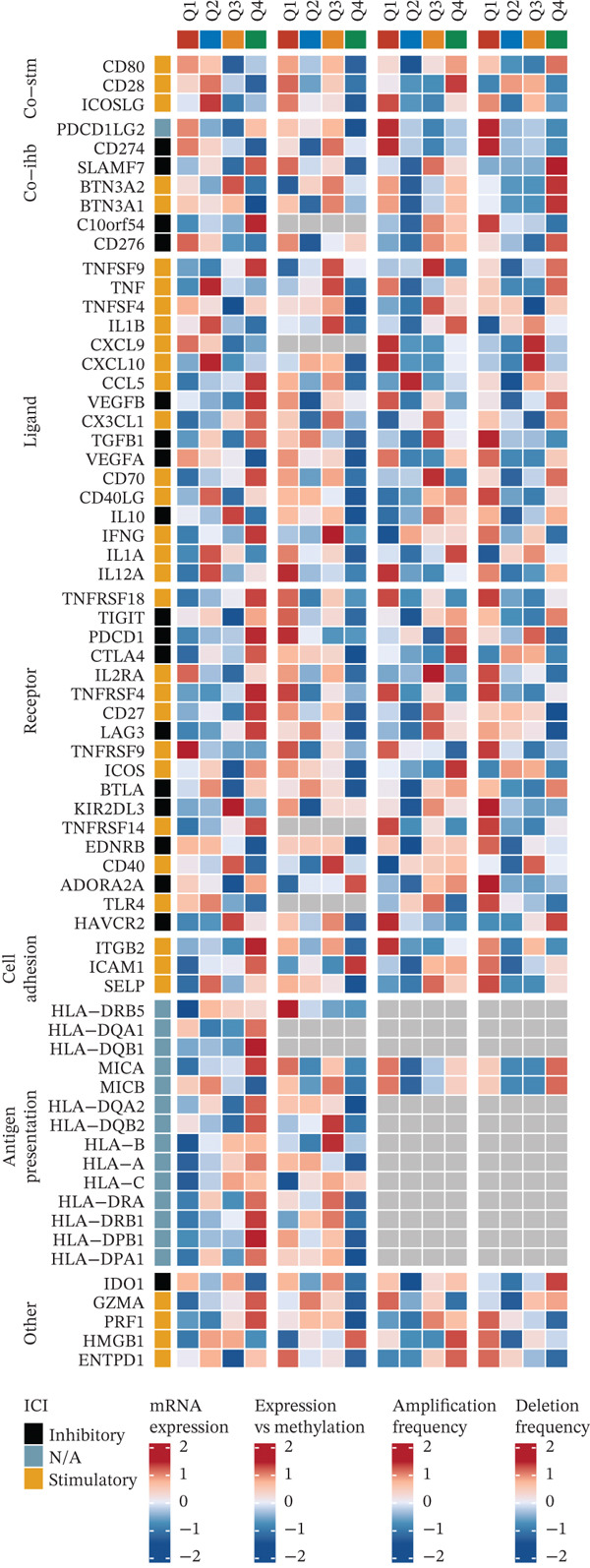
(a)

**Figure:**
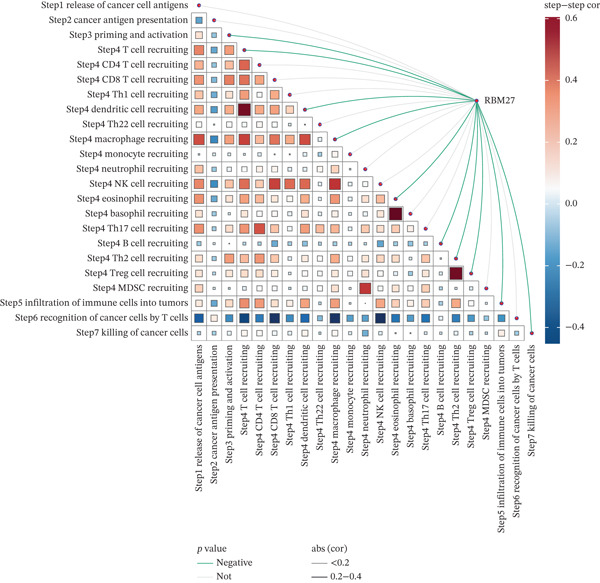
(b)

**Figure:**
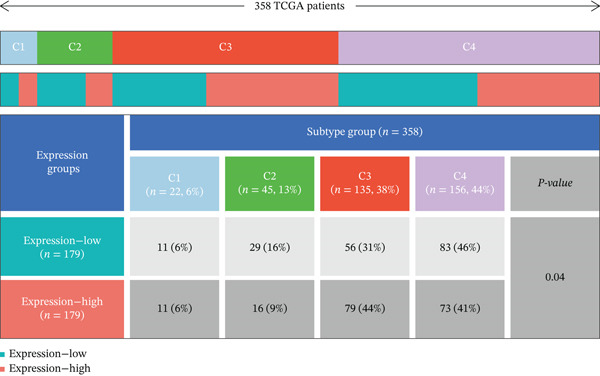
(c)

**Figure:**
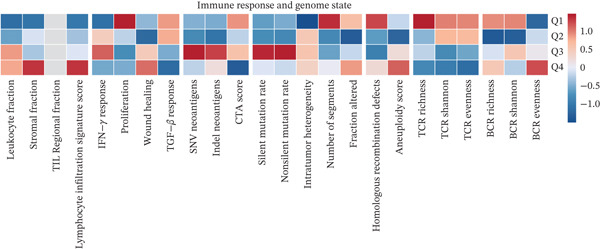
(d)

Immune subtype analysis confirms a significant redistribution in RBM27‐high tumors (*χ* ^2^ test, *p* = 0.04), featuring marked depletion of immunoreactive C2 (IFN‐*γ*‐dominant) subtypes (9% vs. 16% in RBM27‐low) with concomitant enrichment in C3 (inflammatory) subtypes (44% vs. 31%). This redistribution occurs despite C4 (lymphocyte‐depleted) subtypes maintaining dominance in both groups. The selective reduction in C2—characterized by robust CD8^+^ T cell signaling and M1 macrophage polarization—alongside expansion of C3 (which exhibits impaired tumor growth control despite elevated Th17/Th1 signals) (Figure 6d).

### 3.7. Spatial Transcriptomics Validates RBM27 Enrichment in Malignant Niches and Immune Exclusion

Moreover, spatial transcriptomic profiling across four HCC datasets unequivocally demonstrated predominant RBM27 enrichment within malignant tumor regions (Figures 7a, 7b, 7e, 7f, 7i, 7j, 7m, and 7n). Our analysis revealed a consistent spatial pattern wherein elevated RBM27 expression significantly correlated with increased malignant cell density across tissue microregions. This spatial correlation exhibited an inverse relationship with stromal and immune cell infiltration, particularly evident in lymphoid populations (Figures 7c, 7g, 7k, and 7o). Quantitative spatial analysis further confirmed significantly higher RBM27 expression in tumor regions compared with adjacent normal tissues (Figures 7d, 7h, 7l, and 7p). These results collectively underscore RBM27′s critical role in shaping the HCC microenvironment through its malignant cell‐specific expression and association with immune exclusion. Consistent with prior single‐cell correlation analyses, spatial transcriptomics established significant positive correlations between RBM27 and multiple proliferation‐associated genes across solid tumors. This spatial concordance reinforces that RBM27 expression and proliferation markers may be coregulated or functionally synergistic in driving tumor cell proliferation within the HCC ecosystem (Figure 7q).

Figure 7Spatial validation of RBM27‐malignant niche association and immune exclusion. (a, b, e, f, i, j, m, n) Spatial mapping of RBM27 enrichment within malignant regions across four HCC datasets. (c, g, k, o) Spearman′s correlation between cell content and cell content and between cell content and gene expression in all spots. (d, h, l, p) Quantitative comparison of RBM27 expression in tumor versus adjacent normal tissues. (q) Spatial correlation between RBM27 and proliferation‐associated genes (PCNA, Ki67, CENPF, and TOP2A).

**Figure:**
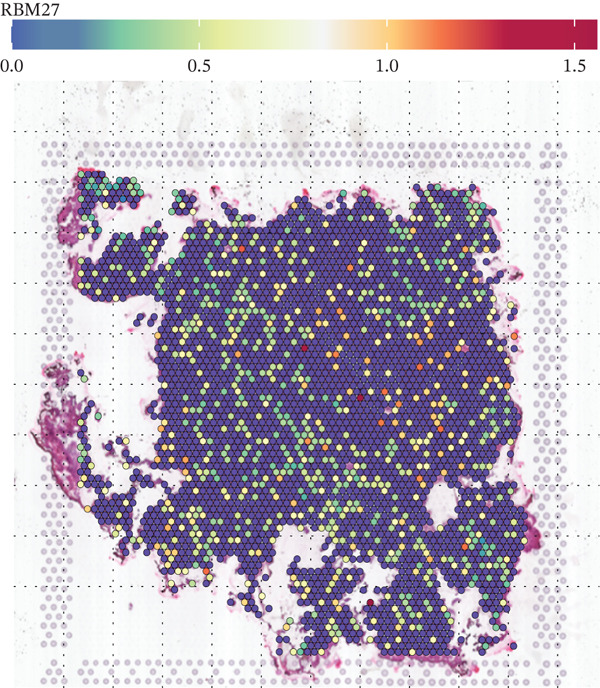
(a)

**Figure:**
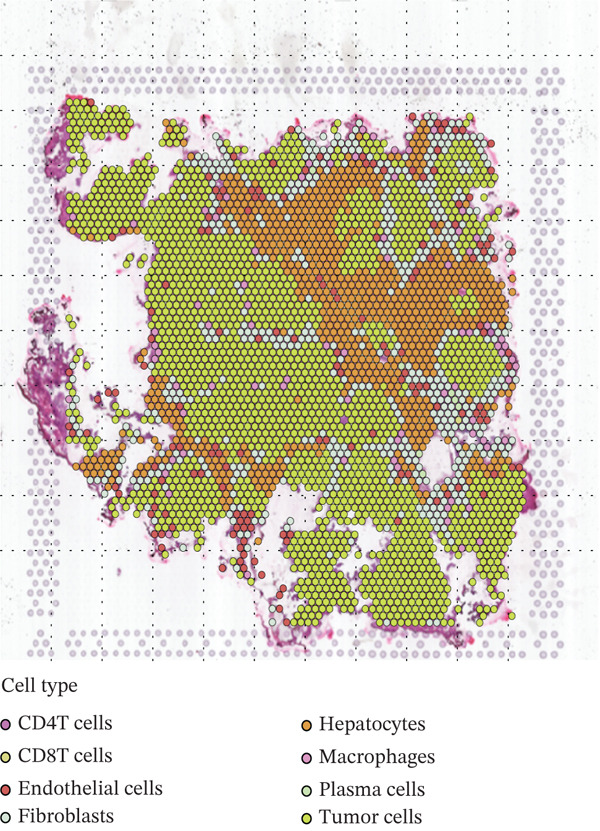
(b)

**Figure:**
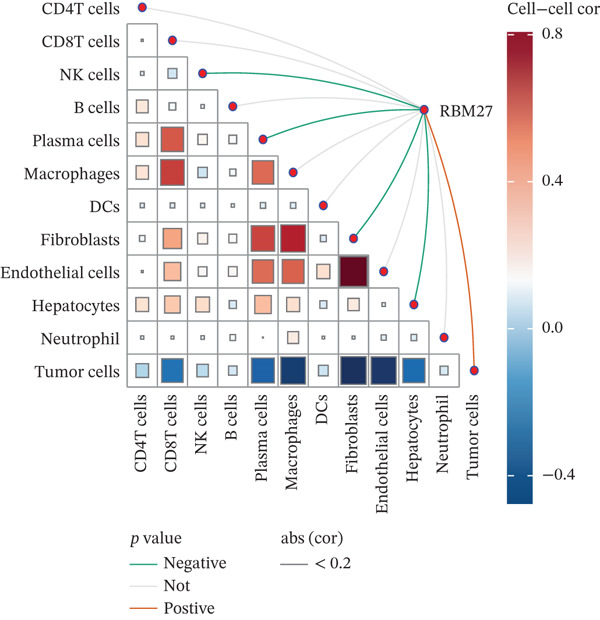
(c)

**Figure:**
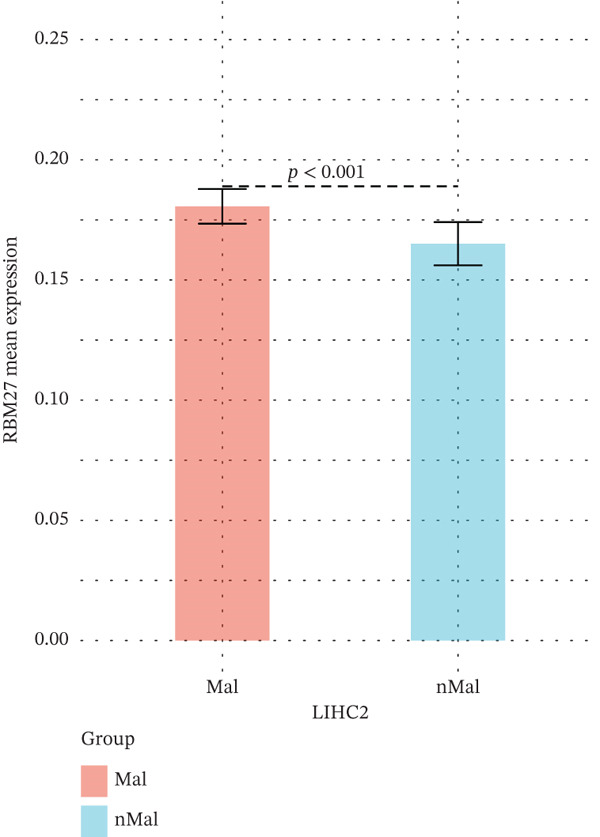
(d)

**Figure:**
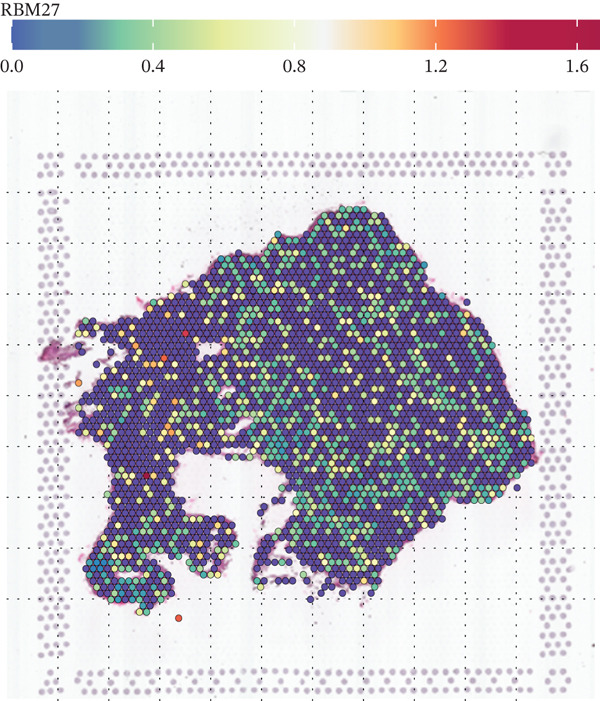
(e)

**Figure:**
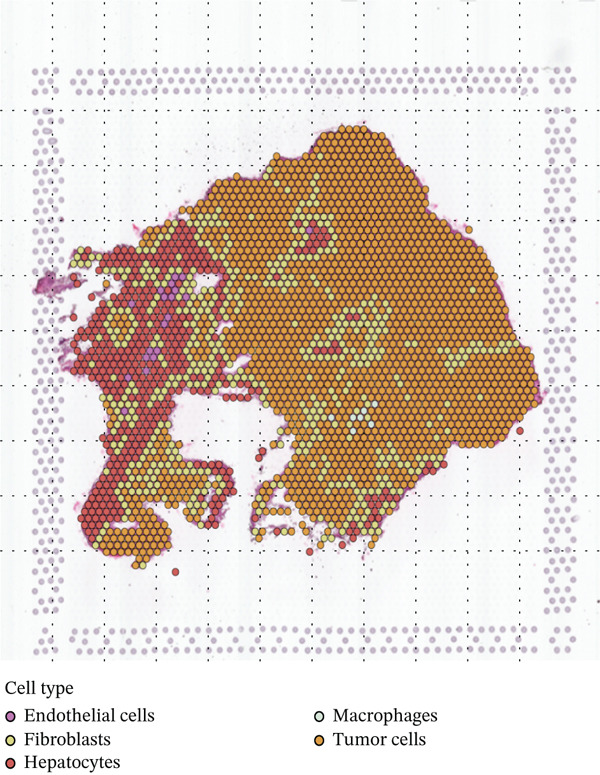
(f)

**Figure:**
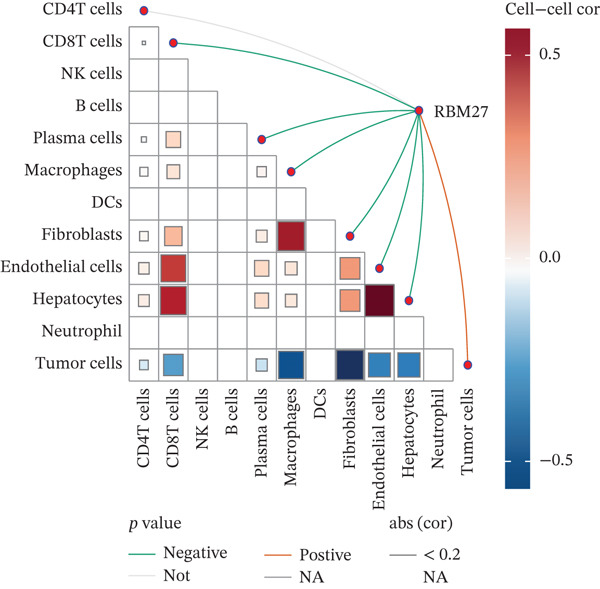
(g)

**Figure:**
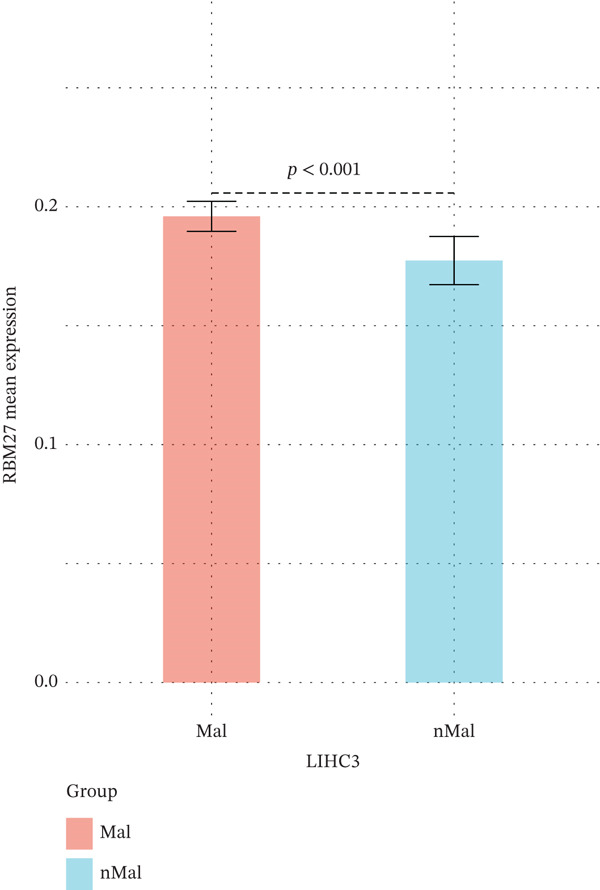
(h)

**Figure:**
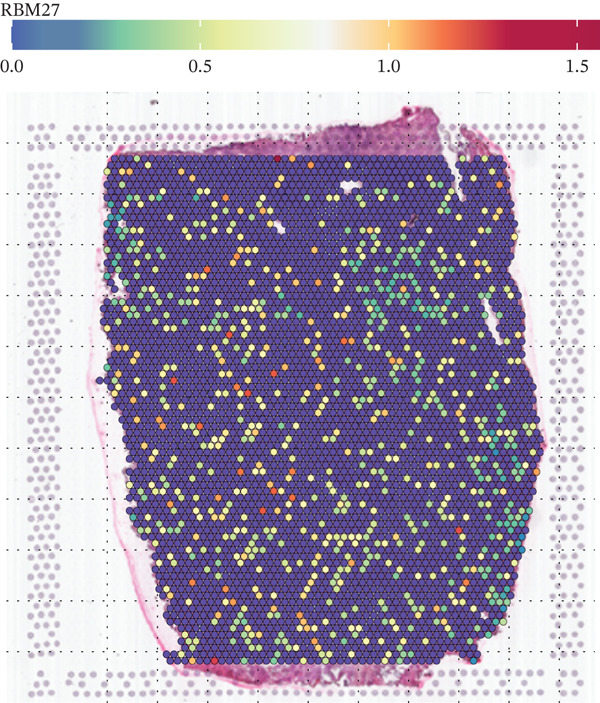
(i)

**Figure:**
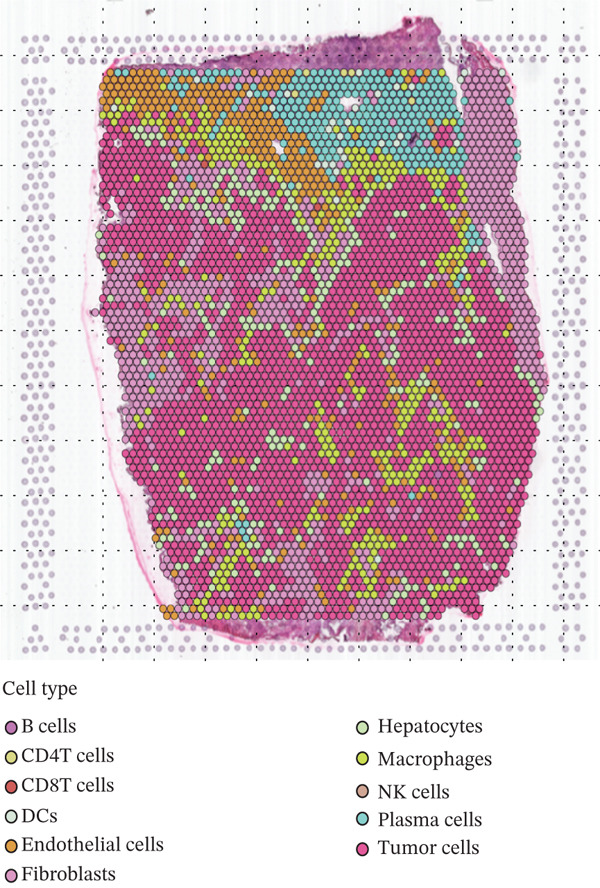
(j)

**Figure:**
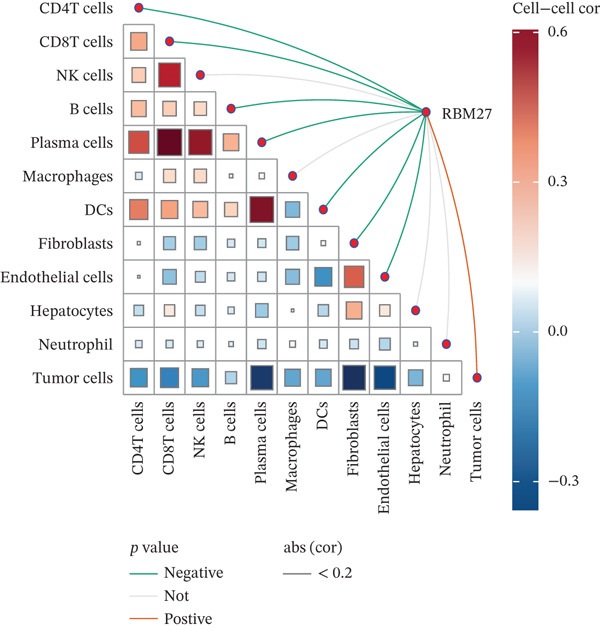
(k)

**Figure:**
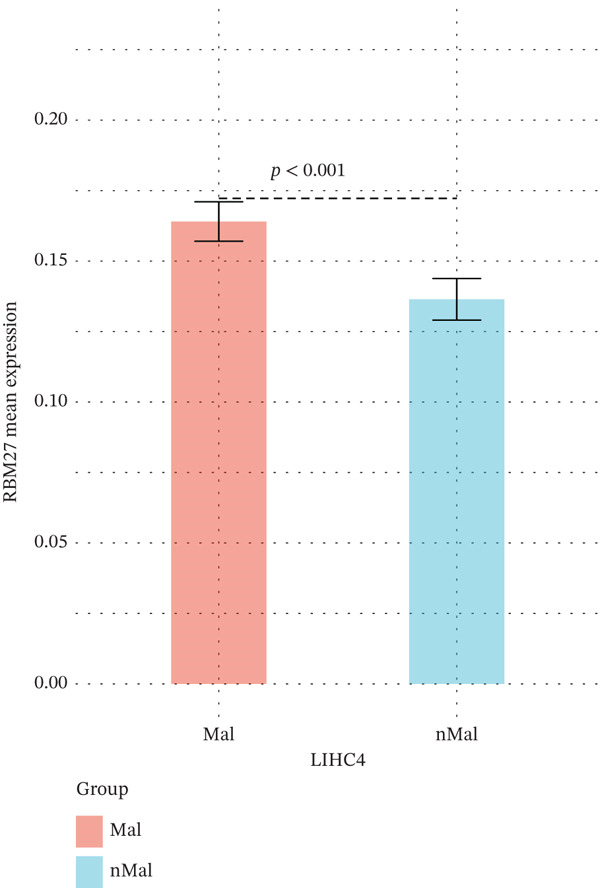
(l)

**Figure:**
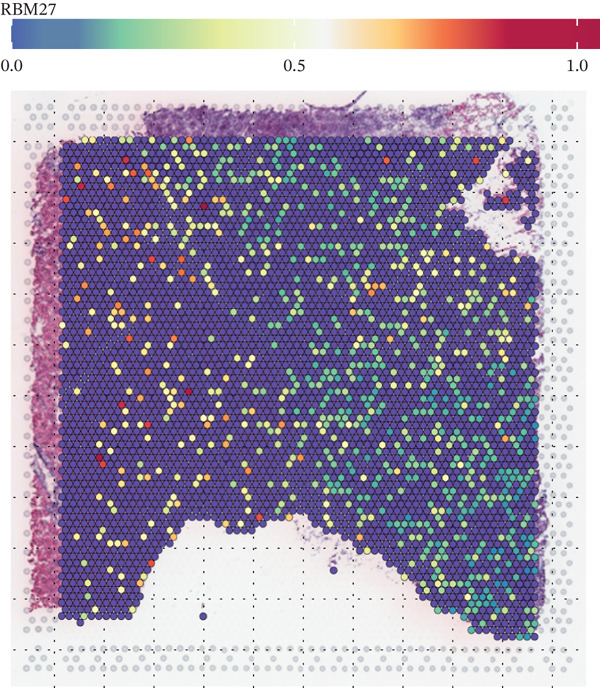
(m)

**Figure:**
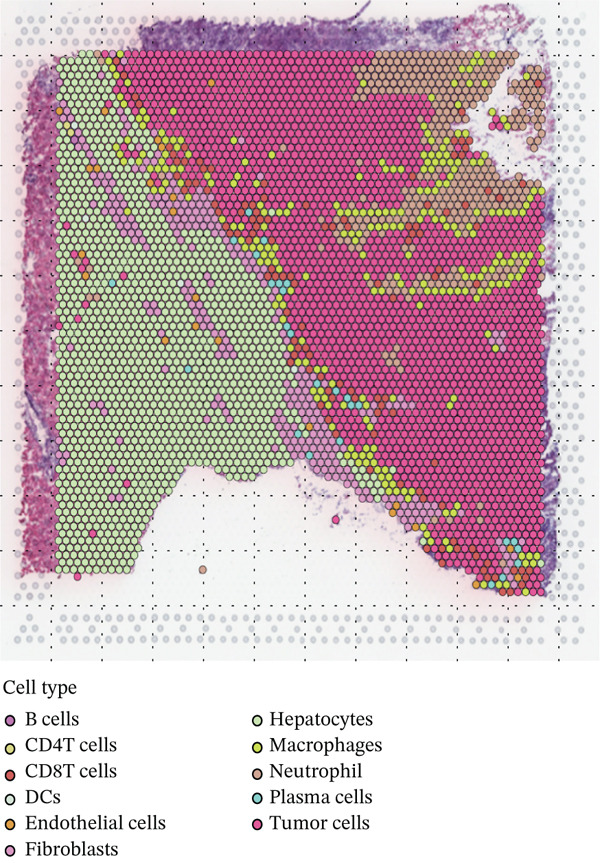
(n)

**Figure:**
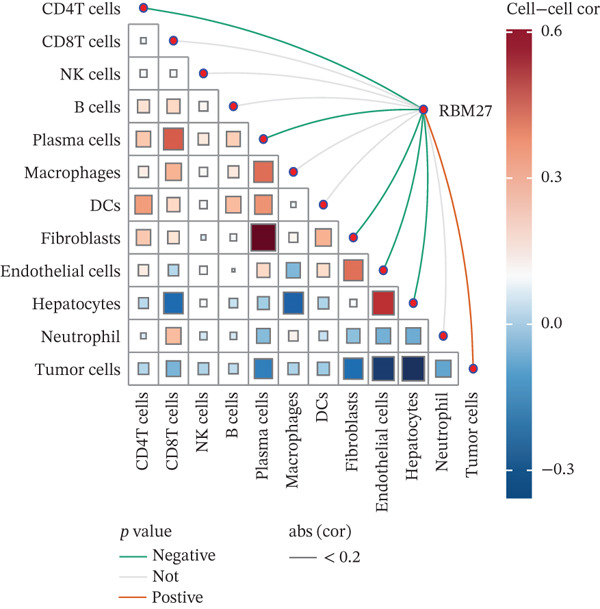
(o)

**Figure:**
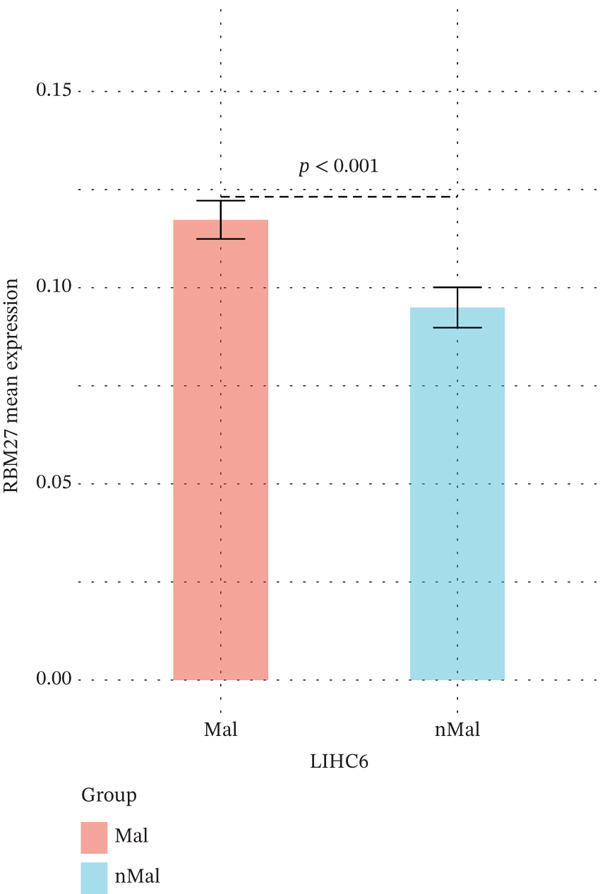
(p)

**Figure:**
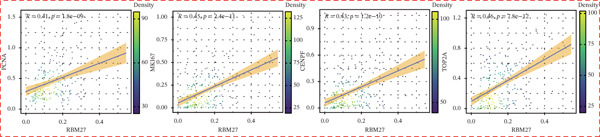
(q)

### 3.8. RBM27‐Related Signaling Pathways Identified Through GO, KEGG, and GSEA Enrichment Analyses

A total of 548 significant DEGs were identified, with 389 upregulated in the high‐RBM27 expression group and 159 downregulated (Figure 8a). GO enrichment analysis demonstrated that DEGs associated with RBM27 were mainly implicated in organelle fission, nuclear division, mitochondrial inner membrane function, mitochondrial matrix processes, electron transfer activity, and primary active transmembrane transporter activity (Figure 8b). KEGG pathway enrichment analysis showed that RBM27‐correlated DEGs were markedly enriched within pathways linked to thermogenesis, OXPHOS, diabetic cardiomyopathy, Huntington′s disease, nonalcoholic fatty liver disease, Parkinson′s disease, chemical carcinogenesis, and reactive oxygen species metabolism (Figure 8c,d). Further analysis using GSEA demonstrated significant enrichment of OXPHOS pathways in both GO and KEGG datasets (Figure 8e,f).

Figure 8Functional enrichment analyses of RBM27‐relatedgenes in HCC. (a) The volcano plot shows the DEGs associated with RBM27. Red: upregulated DEGs, *n* = 389; blue: downregulated DEGs, *n* = 159; gray dots represent nonsignificant DEGs (*p* > 0.05, |log2FC| < 1). (b) The enriched terms in GO categories in HCC. (c, d) KEGG pathway analysis based on RBM27‐associated DEGs. (e, f) GSEA enrichment analysis was used to analyze the pathways shared by GO and KEGG.

**Figure:**
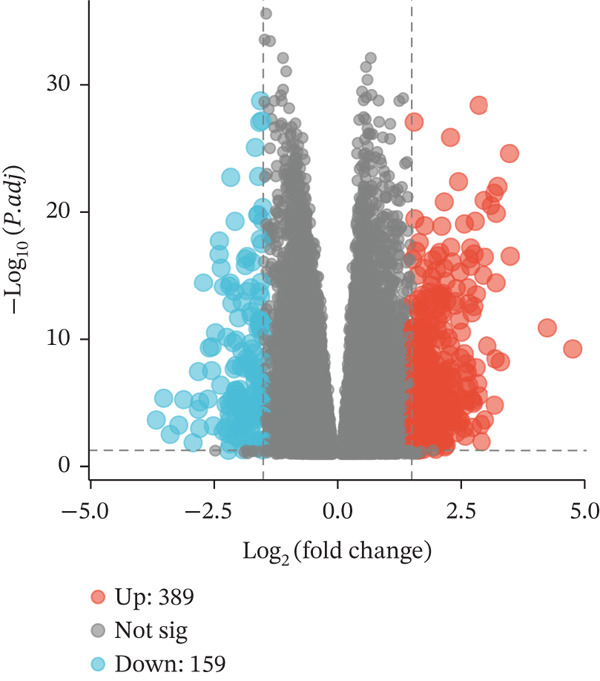
(a)

**Figure:**
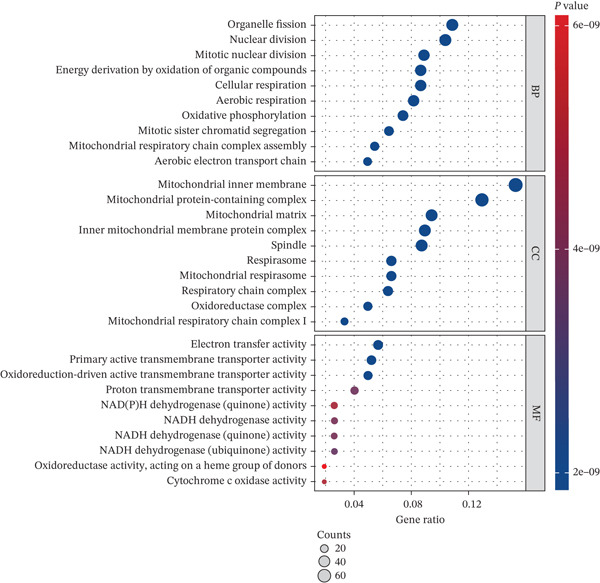
(b)

**Figure:**
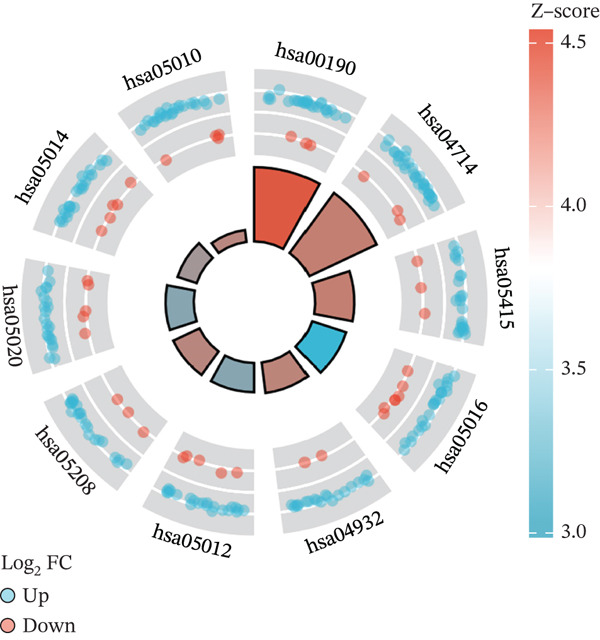
(c)

**Figure:**
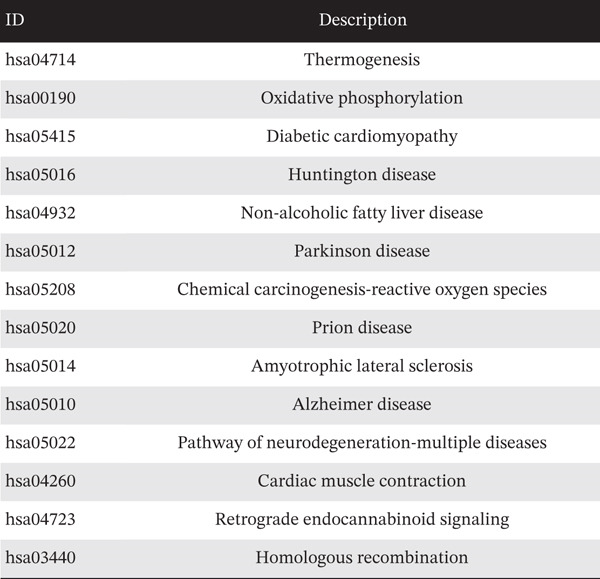
(d)

**Figure:**
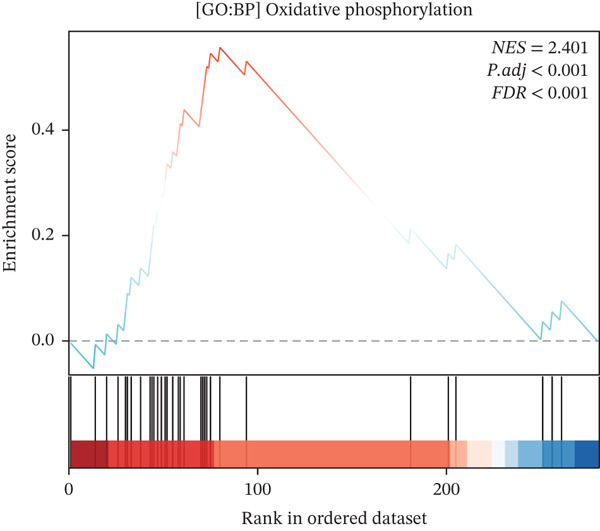
(e)

**Figure:**
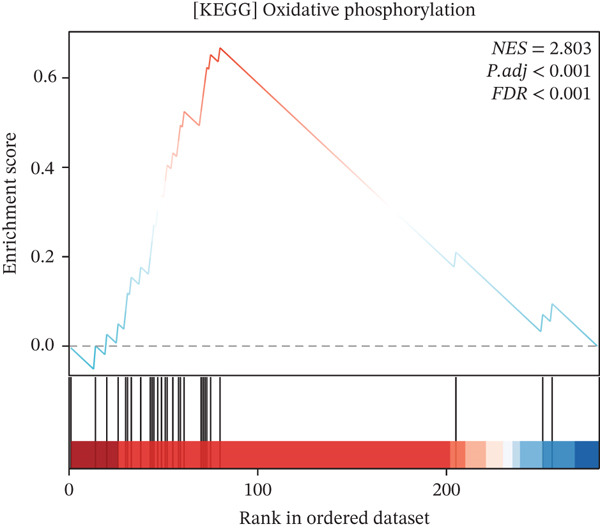
(f)

### 3.9. RBM27 Knockdown Inhibits the Proliferation, Migration and Invasion of HCC

To further explore the biological function of RBM27 in HCC, we adopted lentiviral vectors to downregulate RBM27 expression in HCCLM3 and Huh7 cells. These two cell lines show relatively high levels of RBM27. Western blotting assay verified the efficiency of RBM27 downregulation (Figure 9a). Colony formation assays indicated that RBM27 downregulation remarkably impaired the proliferative activity of HCCLM3 and Huh7 cells, after RBM27 knockdown, the colony formation rate of HCCLM3 cells was reduced by 67.3*%* ± 3.1*%* (sh# 1) and 52.7*%* ± 2.8*%* (sh#2). The colony formation rate of Huh7 cells was decreased by 59.5*%* ± 2.5*%* (sh# 1) and 45.2*%* ± 3.3*%* (sh#2) (*p* < 0.05) (Figure 9b,c). A subcutaneous tumor model was established by injecting shRBM27‐HCCLM3 and control cells into nude mice. RBM27‐knockdown tumors exhibited significantly lower tumor weight and smaller tumor volume than controls (Figures 9d, 9e, and 9f). Transwell assays demonstrated that RBM27 knockdown markedly reduced the migration and invasion capabilities of HCCLM3 (Figure 9g) and Huh7 cells (Figure 9h). Wound healing assays further confirmed that RBM27 suppression impaired cell migration in both HCCLM3 and Huh7 cells (Figure 9i).

Figure 9RBM27 promotes the proliferation, migration, and invasion of HCC in vitro and in vivo. (a) Western blot analysis of RBM27 expression in HCCLM3 and Huh7 cells (shRBM27 and control shCon). (b, c) The proliferation of HCCLM3 and Huh7 cells was detected by colony formation assay. (d) Representative image of nude mice with exfoliated tumors after subcutaneous injection of HCCLM3 cells (*n* = 6). (e) Measurement of tumor weight in RBM27 knockdown nude mice. (f) Tumor volume curves were plotted according to tumor diameter. (g–i) HCCLM3 and Huh7 cells were used to determine cell invasion and migration by Transwell and wound‐healing assays. Scale bars: 50 *μ*m (200×). All experiments were independently repeated three times, and the results were expressed as mean ± standard deviation (SD).  ^∗^ *p* < 0.05,  ^∗∗^ *p* < 0.01,  ^∗∗∗^ *p* < 0.001.

**Figure:**
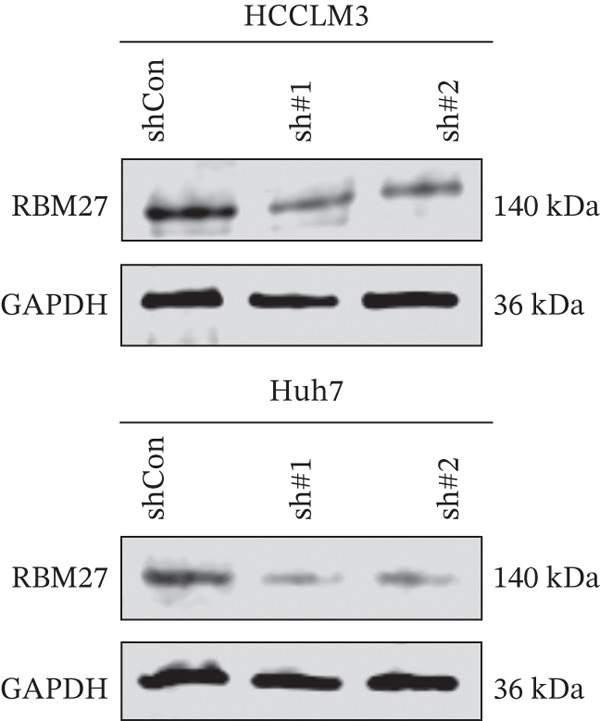
(a)

**Figure:**
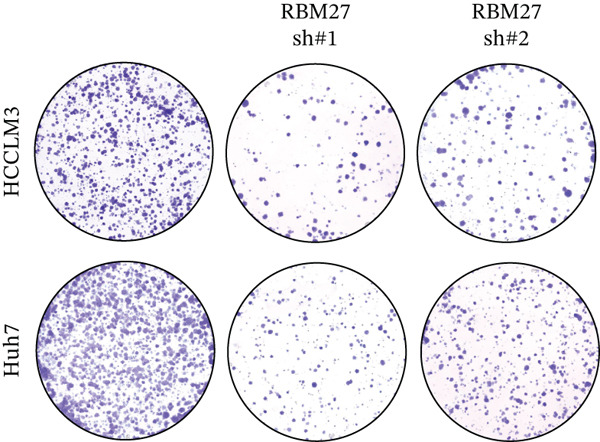
(b)

**Figure:**
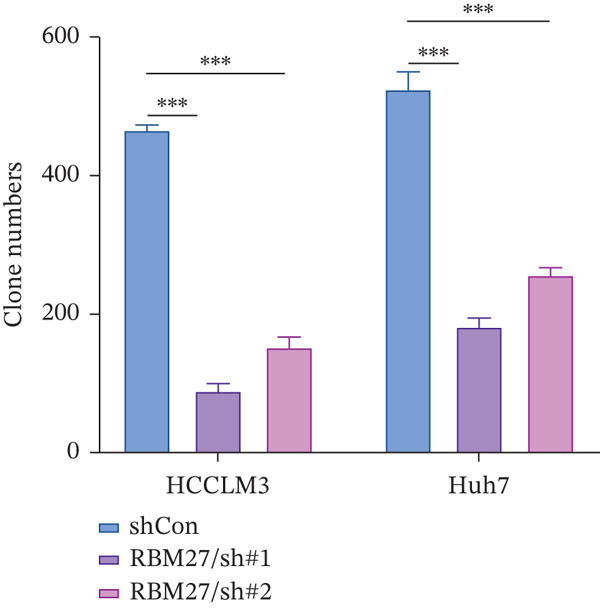
(c)

**Figure:**
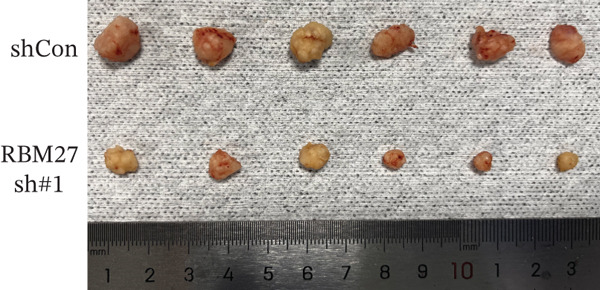
(d)

**Figure:**
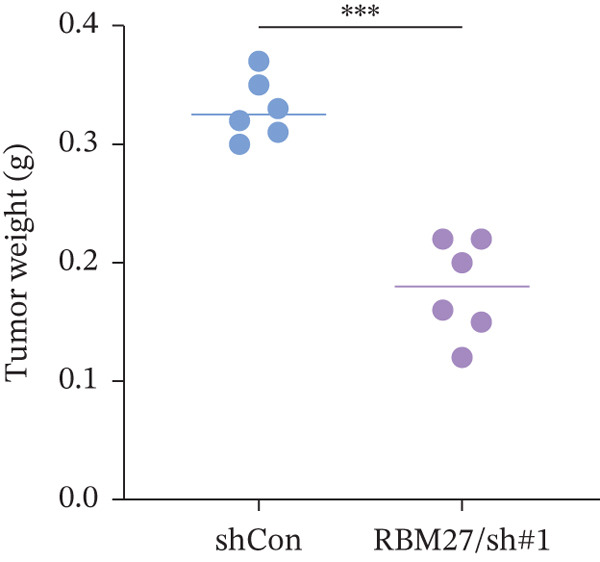
(e)

**Figure:**
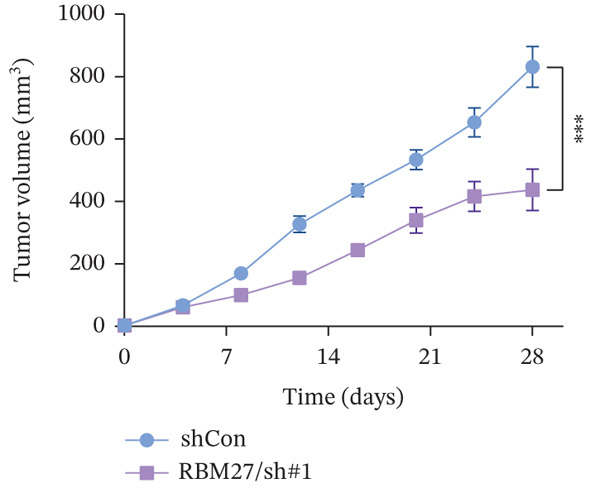
(f)

**Figure:**
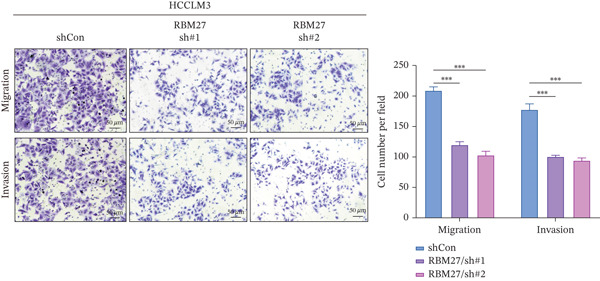
(g)

**Figure:**
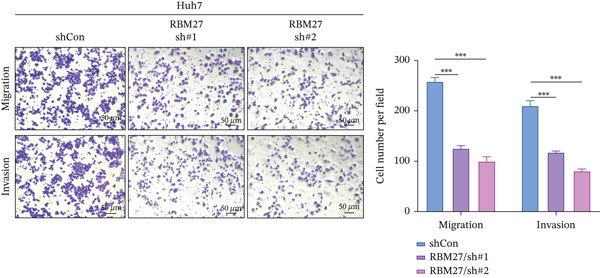
(h)

**Figure:**
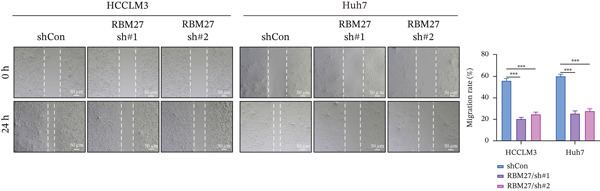
(i)

## 4. Discussion

This study, through comprehensive multiomics analysis and functional validation, demonstrates that RNA‐binding motif protein 27 (RBM27) acts as a master regulator of immune suppression in HCC. RBM27 is significantly upregulated in HCC tissues, and its high expression is closely related to advanced tumor progression, elevated AFP level, and poor prognosis of patients. At the mechanistic level, RBM27 drives metabolic reprogramming through the activation of OXPHOS pathway, and then remodels the TME to form an immunosuppressive state. On the one hand, RBM27 functionally promotes tumor cell proliferation, migration, invasion, and angiogenesis. On the other hand, it significantly changes the immune pattern, depleting cytotoxic lymphocytes and dendritic cells, while enriching regulatory T cells (Tregs) and Th2 cells. Spatial transcriptomes further revealed that RBM27^+^ malignant cells form specific niches characterized by lymphocyte rejection. Together, these effects lead to tumor immune escape, which ultimately promotes HCC progression and poor clinical outcome.

Members of the RBM protein family exhibit diverse functions in various cancers. For instance, low expression of RBM4 in gastric cancer correlates with poor prognosis [9], whereas high expression of RBM15 in colorectal cancer is associated with a favorable prognosis [10]. In contrast, RBM27 is significantly upregulated in HCC, and its high expression correlates with reduced OS, DSS, and PFI in patients with HCC [13, 14]. This study reveals the oncogenic role of RBM27 in HCC, highlighting that its overexpression is independently associated with shorter patient survival. These findings not only expand the functional repertoire of the RBM protein family in tumors but also suggest that RBM27 may promote HCC progression through a unique molecular mechanism. For example, RBM15, as a component of the m6A methyltransferase complex, influences the TME through epigenetic regulation [21].

The genetic alteration of RBM27 in pan‐cancer shows a clear tissue‐specific pattern. Although its overall genomic mutation rate is low (2.5%), its mutation frequency is significantly increased in specific cancers such as endometrial cancer (10.02%) and HCC (1.34%), suggesting its background dependence. In the mutation spectrum, missense mutations dominated (165/232), indicating that the oncogenic potential of RBM27 may mainly result from gain‐of‐function changes of its encoded protein rather than complete inactivation. CNV analysis further revealed the key mechanism of RBM27 dysregulation. The data showed significantly more gene amplifications (detected in nearly one‐third of the samples) than gene deletions (9% of the samples). This amplification advantage is consistent with the RBM27 overexpression phenomenon observed in HCC and strongly supports the CNV‐mediated gene dosage increase as an important molecular basis driving its expression upregulation. Combined with functional studies showing that knockdown of RBM27 inhibits HCC proliferation and metastasis, these evidences together suggest that RBM27 may act as a dose‐dependent oncogene.

As an RBP, RBM27 likely participates in the regulation of RNA metabolism through interactions with other proteins such as ZC3H18. ZC3H18 is an RBP known to be involved in mRNA stability and translational regulation. The high correlation score between RBM27 and ZC3H18 indicates a potential synergistic effect between the two in RNA metabolism. The predicted interaction sites provided by Alphafold3 offer a theoretical basis for further experimental validation [22]. GO enrichment analysis indicated that genes interacting with RBM27 are involved in mRNA processing, RNA splicing, and regulation of mRNA metabolism, processes known to play important roles in cancer development [23–25]. These findings are consistent with the function of RBM27 as an RBP and suggest that it may play a pivotal role in posttranscriptional regulation.

Meanwhile, in vitro experiments demonstrated that RBM27 knockdown significantly inhibited the proliferation, migration, and invasion of HCCLM3 and Huh7 cells in vitro. In animal models, RBM27 knockdown resulted in markedly reduced tumor volume and weight, highlighting its crucial role in tumor growth. Mechanistically, GO/KEGG enrichment analyses revealed that RBM27‐associated differential genes were significantly enriched in the OXPHOS pathway. The result is that cells depend on OXPHOS to maintain energy metabolism and malignant phenotype in hypoxic microenvironment. For instance, pancreatic cancer cells can survive after oncogene ablation by enhancing OXPHOS [26]. Our study suggests that RBM27 may help HCC cells adapt to metabolic stress by regulating the stability or translation efficiency of RNA associated with mitochondrial metabolism. However, whether RBM27 directly binds to the mRNA of OXPHOS genes (e.g., NDUFB8, SDHA, UQCRQ, and ATP5ME) or exerts indirect effects through mitochondrial RNA processing remains to be verified using techniques such as RIP‐seq or ChIP‐seq.

We demonstrate that RBM27‐driven OXPHOS activation directly fuels an immunosuppressive microenvironment. This metabolic shift depletes cytotoxic lymphocytes and dendritic cells while enriching for Tregs/Th2 cells, thereby impairing the cancer‐immunity cycle at multiple steps. The spatial exclusion of lymphoid populations within RBM27^+^ malignant niches provides histological validation of this immune‐cold phenotype, consistent with the enrichment of C3 inflammatory subtypes in RBM27‐high tumors. This indicates that RBM27 may promote immune evasion in HCC by suppressing antitumor immune responses, a phenomenon that likely involves several mechanisms—including the secretion of immunosuppressive factors [27, 28]. RBM27 may promote M2 macrophage polarization and Treg recruitment by regulating factors such as IL‐10, TGF‐*β*, or VEGF. For example, the activation of OXPHOS enhances lactate secretion by tumor cells, which acidifies the TME and inhibits T cell function [29]. Additionally, RBM27 may influence antigen presentation by downregulating MHC‐I or antigen processing genes such as PSMB8, leading to reduced tumor antigen presentation and immune surveillance escape [30]. Given that RBM27‐high HCC exhibits immune exclusion (lymphocyte depletion, Treg/Th2 enrichment) and impaired cancer‐immunity cycle, we speculate that high RBM27 expression may predict resistance to PD‐1/PD‐L1 inhibitors. This is consistent with previous studies showing that immune‐cold tumors (low T cell infiltration) often respond poorly to checkpoint inhibitors. Future clinical studies could validate whether RBM27 can serve as a predictive biomarker for immunotherapy efficacy, and combining RBM27 inhibition with PD‐1/PD‐L1 blockers may reverse immune exclusion and enhance therapeutic response.

This study has several limitations. First, the clinical validation cohort for RBM27 expression (20 pairs of HCC and adjacent nontumor tissues) is relatively small; future studies with larger multicenter cohorts are needed to confirm its diagnostic and prognostic value. Second, we only explored the role of RBM27 knockdown in vitro and in vivo, and did not test specific inhibitors. Third, the molecular mechanism by which RBM27 regulates OXPHOS requires further validation via RIP‐seq or ChIP‐seq.

## 5. Conclusions

Based on multiomics and functional validation, RBM27 drives HCC progression by activating OXPHOS to remodel an immunosuppressive TME, deplete cytotoxic lymphocytes, enrich Tregs/Th2 cells, and promote tumor growth/metastasis, serving as a prognostic biomarker and potential therapeutic target, providing a theoretical basis for developing targeted therapies against HCC immune escape.

## Author Contributions

W.G. and D.C. conducted the experiments and analyzed the data. X.C., X.W., and W.L. contributed to the technical assistance and discussion. T.D., X.Z., D.W., and C.H. are the guarantors of this work. Wenjuan Gao and Dongwei Cong are joint first authors and contributed equally to this research.

## Funding

No funding was received for this manuscript.

## Ethics Statement

This research obtained approval from the Ethics Committee of the 962nd Hospital of the Chinese People′s Liberation Army (Ethical Approval No. 2024‐01) and was carried out in line with the tenets of the Declaration of Helsinki. All participants submitted written informed consent prior to their involvement in the research.

## Conflicts of Interest

The authors declare no conflicts of interest.

## Supporting Information

Additional supporting information can be found online in the Supporting Information section.

## Supporting information


**Supporting Information 1** Figure S1: (a) Comparison of RBM27 expression levels in different cancer tissues and normal tissues. (b) The expression levels of RBM27 in different paired cancer tissues and normal tissues were compared.(c–f) GEO databases were used to analyze the expression of RBM27 in HCC tissues.

**Supporting Information 2** Table S1: Characteristics of patients with HCC in TCGA.

**Supporting Information 3** Table S2: Correlation between clinicopathological variables and RBM27 expression.

**Supporting Information 4** “Research Guidelines for Genetic/Molecular Disease Studies” (Version 2024) is include in supporting information file.

## Data Availability

The data that support the findings of this study are available from the corresponding authors upon reasonable request.
