# Supplementary figures and images for: Multimodal Analysis Reveals Immune Suppression Associated With Hepatocellular Carcinoma Related to RBM27 and Constructs a Prognostic Model
Source: Hum Mutat. 2026 Mar 23;2026:4343678. doi: 10.1155/humu/4343678 (PMC13369010; doi:10.1155/humu/4343678)

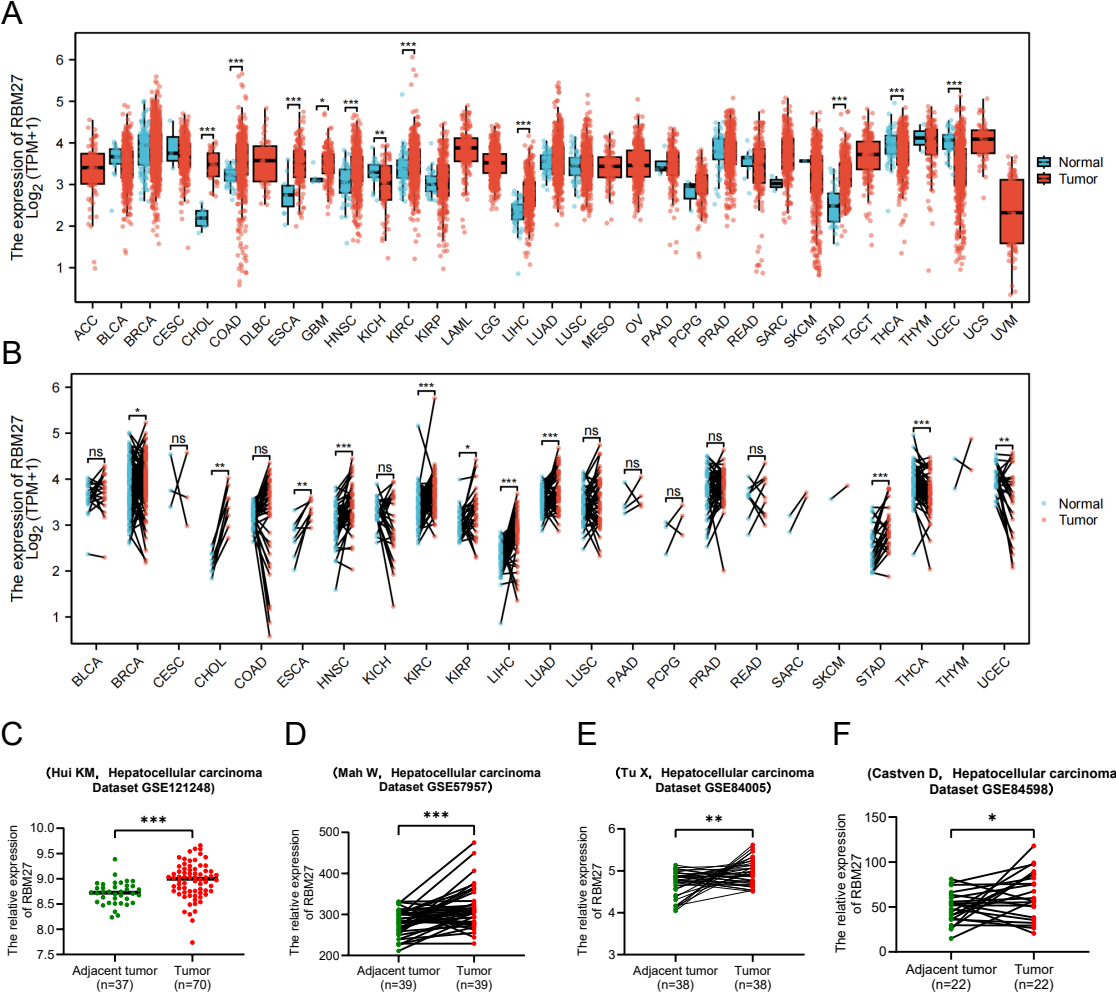

Supplement: Supplementary file 1 — Supporting Information 1 Figure S1: (a) Comparison of RBM27 expression levels in different cancer tissues and normal tissues. (b) The expression levels of RBM27 in different paired cancer tissues and normal tissues were compared.(c–f) GEO databases were used to analyze the expression of RBM27 in HCC tissues. [file HUMU-2026-4343678-s001.pdf]
