# Supplementary material for: Multimodal Analysis Reveals Immune Suppression Associated With Hepatocellular Carcinoma Related to RBM27 and Constructs a Prognostic Model
Source: Hum Mutat. 2026 Mar 23;2026:4343678. doi: 10.1155/humu/4343678 (PMC13369010; doi:10.1155/humu/4343678)
Supplement: Supplementary file 2 — Supporting Information 2 Table S1: Characteristics of patients with HCC in TCGA. [file HUMU-2026-4343678-s004.docx]

**Characteristics of patients with HCC in TCGA**

| Characteristic | levels | Overall |
| --- | --- | --- |
| n |  | 374 |
| Gender, n (%) | Female | 121 (32.4%) |
|  | Male | 253 (67.6%) |
| Race, n (%) | Asian | 160 (44.2%) |
|  | Black or African American | 17 (4.7%) |
|  | White | 185 (51.1%) |
| Age, n (%) | <=60 | 177 (47.5%) |
|  | >60 | 196 (52.5%) |
| T stage, n (%) | T1 | 183 (49.3%) |
|  | T2 | 95 (25.6%) |
|  | T3 | 80 (21.6%) |
|  | T4 | 13 (3.5%) |
| N stage, n (%) | N0 | 254 (98.4%) |
|  | N1 | 4 (1.6%) |
| M stage, n (%) | M0 | 268 (98.5%) |
|  | M1 | 4 (1.5%) |
| Pathologic stage, n (%) | Stage I | 173 (49.4%) |
|  | Stage II | 87 (24.9%) |
|  | Stage III | 85 (24.3%) |
|  | Stage IV | 5 (1.4%) |
| Tumor status, n (%) | Tumor free | 202 (56.9%) |
|  | With tumor | 153 (43.1%) |
| Residual tumor, n (%) | R0 | 327 (94.8%) |
|  | R1 | 17 (4.9%) |
|  | R2 | 1 (0.3%) |
| Histologic grade, n (%) | G1 | 55 (14.9%) |
|  | G2 | 178 (48.2%) |
|  | G3 | 124 (33.6%) |
|  | G4 | 12 (3.3%) |
| AFP(ng/ml), n (%) | <=400 | 215 (76.8%) |
|  | >400 | 65 (23.2%) |
