# Supplementary material for: Multimodal Analysis Reveals Immune Suppression Associated With Hepatocellular Carcinoma Related to RBM27 and Constructs a Prognostic Model
Source: Hum Mutat. 2026 Mar 23;2026:4343678. doi: 10.1155/humu/4343678 (PMC13369010; doi:10.1155/humu/4343678)
Supplement: Supplementary file 3 — Supporting Information 3 Table S2: Correlation between clinicopathological variables and RBM27 expression. [file HUMU-2026-4343678-s003.docx]

**Correlation between clinicopathological variables and RBM27 expression**

| Characteristics | Low expression of RBM27 | High expression of RBM27 | P value |
| --- | --- | --- | --- |
| n | 187 | 187 |  |
| Pathologic T stage, n (%) |  |  | 0.093 |
| T1 | 100 (27%) | 83 (22.4%) |  |
| T2 | 49 (13.2%) | 46 (12.4%) |  |
| T3 | 31 (8.4%) | 49 (13.2%) |  |
| T4 | 5 (1.3%) | 8 (2.2%) |  |
| Pathologic N stage, n (%) |  |  | 0.693 |
| N0 | 121 (46.9%) | 133 (51.6%) |  |
| N1 | 1 (0.4%) | 3 (1.2%) |  |
| Pathologic M stage, n (%) |  |  | 0.635 |
| M0 | 133 (48.9%) | 135 (49.6%) |  |
| M1 | 1 (0.4%) | 3 (1.1%) |  |
| Pathologic stage, n (%) |  |  | 0.063 |
| Stage I | 93 (26.6%) | 80 (22.9%) |  |
| Stage II | 48 (13.7%) | 39 (11.1%) |  |
| Stage III | 32 (9.1%) | 53 (15.1%) |  |
| Stage IV | 2 (0.6%) | 3 (0.9%) |  |
| **Tumor status, n (%)** |  |  | **0.040** |
| Tumor free | 112 (31.5%) | 90 (25.4%) |  |
| With tumor | 68 (19.2%) | 85 (23.9%) |  |
| Gender, n (%) |  |  | 0.097 |
| Female | 53 (14.2%) | 68 (18.2%) |  |
| Male | 134 (35.8%) | 119 (31.8%) |  |
| Age, n (%) |  |  | 0.275 |
| <= 60 | 83 (22.3%) | 94 (25.2%) |  |
| > 60 | 103 (27.6%) | 93 (24.9%) |  |
| **Weight, n (%)** |  |  | **0.016** |
| <= 70 | 83 (24%) | 101 (29.2%) |  |
| > 70 | 94 (27.2%) | 68 (19.7%) |  |
| Height, n (%) |  |  | 0.138 |
| < 170 | 97 (28.4%) | 104 (30.5%) |  |
| >= 170 | 79 (23.2%) | 61 (17.9%) |  |
| BMI, n (%) |  |  | 0.131 |
| <= 25 | 85 (25.2%) | 92 (27.3%) |  |
| > 25 | 90 (26.7%) | 70 (20.8%) |  |
| **AFP(ng/ml), n (%)** |  |  | **0.017** |
| <= 400 | 119 (42.5%) | 96 (34.3%) |  |
| > 400 | 25 (8.9%) | 40 (14.3%) |  |
| Residual tumor, n (%) |  |  | 0.172 |
| R0 | 171 (49.7%) | 156 (45.3%) |  |
| R1 | 6 (1.7%) | 11 (3.2%) |  |
| Child-Pugh grade, n (%) |  |  | 0.895 |
| A | 118 (49.2%) | 101 (42.1%) |  |
| B | 11 (4.6%) | 10 (4.2%) |  |
| Vascular invasion, n (%) |  |  | 0.870 |
| No | 106 (33.3%) | 102 (32.1%) |  |
| Yes | 55 (17.3%) | 55 (17.3%) |  |
| Albumin(g/dl), n (%) |  |  | 0.285 |
| < 3.5 | 40 (13.3%) | 29 (9.7%) |  |
| >= 3.5 | 117 (39%) | 114 (38%) |  |
| Fibrosis ishak score, n (%) |  |  | 0.337 |
| 0 | 34 (15.8%) | 41 (19.1%) |  |
| 1/2 | 19 (8.8%) | 12 (5.6%) |  |
| 3/4 | 12 (5.6%) | 16 (7.4%) |  |
| 5 | 5 (2.3%) | 4 (1.9%) |  |
| 6 | 42 (19.5%) | 30 (14%) |  |
