# Supplementary material for: Multimodal Analysis Reveals Immune Suppression Associated With Hepatocellular Carcinoma Related to RBM27 and Constructs a Prognostic Model
Source: Hum Mutat. 2026 Mar 23;2026:4343678. doi: 10.1155/humu/4343678 (PMC13369010; doi:10.1155/humu/4343678)
Supplement: Supplementary file 4 — Supporting Information 4 “Research Guidelines for Genetic/Molecular Disease Studies” (Version 2024) is include in supporting information file. [file HUMU-2026-4343678-s002.docx]

Supplementary Materials

**Multimodal analysis reveals immune suppression associated with hepatocellular carcinoma related to RBM27 and constructs a prognostic model**

**Methods and material**

**Integrated RBM27 Immunoprofiling and Cancer Immunity Cycle Analysis**

To elucidate their biological relevance, we systematically analyzed expression profiles, copy number variations, and DNA methylation patterns relative to RBM27 expression levels. RBM27 expression was categorized into quartiles (Q1, Q2, Q3, Q4), with heatmap components arranged left to right. mRNA expression values represent the median of normalized expression levels. Expression-methylation correlations reflect the relationship between RBM27 expression and DNA methylation β-values. Amplification frequency was defined as the difference between subtype-specific and pan-cancer amplification proportions, while deletion frequency followed analogous calculations. Aligning with Thorsson et al.'s immune response framework (1), average scores for each metric were computed per quartile (excluding missing values) and visualized using the pheatmap package. Furthermore, leveraging Thorsson et al.'s immunogenomic analysis of >10,000 tumors, we examined the distribution of six pan-cancer immune subtypes—C1 (wound healing), C2 (IFN-γ dominant), C3 (inflammatory), C4 (lymphocyte depleted), C5 (immunologically quiet), and C6 (TGF-β dominant)—across RBM27-high and RBM27-low cohorts.

The immunological defense against malignancies operates through a coordinated cascade of biological interactions termed the cancer immunity cycle. TIP (Tumor Immunophenotype Profiler) serves as an integrative computational platform that combines two established analytical approaches - ssGSEA and CIBERSORT - to monitor, evaluate, and graphically represent both the tumor immune microenvironment status and immune cell infiltration levels across all seven phases of this immunity cycle using transcriptomic data. This analytical framework examines immune activity throughout each cycle stage: (1) neoantigen liberation, (2) antigen processing and presentation, (3) immune activation priming, (4) leukocyte trafficking to malignant tissue, (5) immune cell penetration into tumors, (6) tumor antigen recognition by T lymphocytes, and (7) malignant cell elimination. The TIP platform generates quantitative metrics for each malignancy and cycle phase. Through nonparametric correlation assessment, we investigated relationships between RBM27 expression patterns and TIP metrics, while examining interconnections among different TIP parameters (2). All association patterns were graphically represented using the linkET visualization toolkit.

**Spatial Transcriptomic Profiling of RBM27 in LIHC**

To map RBM27 expression heterogeneity across malignancies, we leveraged single-cell transcriptomic data from the Tumor Immune Single-cell Hub (TISCH) database (3) ,we interrogated spatial transcriptomic landscapes across solid tumors via the Sparkle database(https://grswsci.top/)—a curated repository integrating 204 high-resolution 10x Genomics Visium slices spanning non-small cell lung, colorectal, breast, and other carcinomas. Leveraging SpatialTME's Cottrazm-based deconvolution pipeline (4,5), Sparkle reconstructs pan-cancer tissue microenvironments by resolving cellular compositions within spatially barcoded regions. Microregion identities were classified according to dominant cell types, enabling heatmap visualization of RBM27 expression across cellular niches. Spearman correlation quantified: (1) pairwise associations between resident cell populations, and (2) relationships between RBM27 abundance and specific cell-type fractions, with linkET package rendering these interdependencies graphically.

Tissue domains were stratified by malignant cell prevalence into two tiers: malignant (Mal) and non-malignant (nMal). Spatial heterogeneity in RBM27 distribution was assessed through Wilcoxon rank-sum tests comparing expression between Mal/nMal compartments, with mean expression plots highlighting topographical variation.

**Patients and Clinical Samples**

Paired HCC specimens and adjacent non-neoplastic liver tissues (n=20) were prospectively collected from patients undergoing curative hepatic resection at the Department of Hepatobiliary Surgery, PLA Joint Logistics Support Force 962 Hospital. Ethical approval for this tissue banking protocol was obtained from the Institutional Review Board of PLA 962 Hospital (Ethical Review.2024.No1). All samples were acquired through standardized surgical procedures performed between February 2021 and June 2024, with written informed consent secured prior to tissue acquisition.

**Cell lines and cell culture**

The human HCC lines MHCC97-H, Huh7, HCCLM3, and PLC/PRF/5 were acquired from Chinese Academy of Sciences (Shanghai), while WRL-68 hepatocytes and HUVECs came from AcceGen (USA). Cells were maintained in DMEM (Gibco) containing 10% FBS, penicillin (100 U/mL), and streptomycin (100 µg/mL) at 37°C with 5% CO₂.

**Western Blotting**

We extracted proteins from cells via radioimmunoprecipitation (RIPA) and separated them via electrophoresis (Beyotime, Shanghai, China). We transferred proteins to NC membranes (Millipore, Billerica, mA, USA) for 100 min using a steady current of 300 mA. The membranes were blocked with rapid blocking solution 20min and samples were treated with the primary antibody at 4°C overnight, then exposed to the secondary antibody for 1.5 hours. Protein visualization and analysis were performed using the Odyssey ® Imaging System (LI-COR, USA). Antibodies against RBM27 were purchased from Proteintech (Cat No. 31850-1-AP); secondary anti-rabbit and **Quantitative reverse transcription-PCR (RT-qPCR)**

Total RNA was extracted using TRIzol reagent and treated with DNase I to degrade any remaining DNA (Ambion). RT-qPCR was carried out on an ABI Prism 7900HT equipment (Life Technologies, Carlsbad, CA, USA) using the Power SYBR Green PCR master kit. The following primers were used: RBM27-F, 5’-TCAACAGTGCACGGAGGTAT-3’;RBM27-R,5’-AGCACCACCAGACTGATTCA-3’;GAPDH-F,5’ -TGACTTCAACAGCGACACCCA-3’ and GAPDH-R, 5’-CACCCTGTTGCTGTAGCCAAA-3’. GAPDH was used as a control to determine changes in mRNA levels using the 2-ΔΔCT method.

**Lentivirus Transfection**

Lentiviral constructs targeting human RBM27 (lenti-RBM27) were acquired from Hanbio Biotechnology (Shanghai). Empty vector controls (lenti-Con and lenti-shCon) served as negative comparators. Transfection was conducted at an MOI of 10–30 using polybrene (5μg/mL). Post-infection, puromycin selection (3.5μg/mL; Sigma-Aldrich) was applied for 14 days to isolate single-cell clones. Stable transfectants were expanded for subsequent in vitro and in vivo studies.

RBM27 lentiviral shRNA sequence design (human)

shRBM27#1:F:5’-CCGGCGGAGATGTTCTACAAGAAGAACTCGAGTTCTTCTTGTAGAACATCTCCGTTTTT-3’

R:5’-AAAAACGGAGATGTTCTACAAGAAGAACTCGAGTTCTTCTTGTAGAACA TCTCCG-3’

shRBM27#2:F:5’-CCGGTTGAGCTGCTGAAGATGAAGACTCGAGTCTTCATCTTCAGCAGC TCAATTTTT-3’

R:5’-AAAAATTGAGCTGCTGAAGATGAAGACTCGAGTCTTCATCTTCAGCAGC TCAACCG-3’

shCon:F:5’-CCGGTTCTCCGAACGTGTCACGTTCTCGAGAACGTGACACGTTCGGAG AATTTTT-3’

R:5’-AAAAATTCTCCGAACGTGTCACGTTCTCGAGAACGTGACACGTTCGGAG AACCG-3’

**Colony formation**

For colony formation assessment, 1000 cells were seeded per 6 cm culture dish and maintained for two weeks. Following incubation, media was removed, colonies were immobilized using 4% paraformaldehyde (PFA), and subsequently dyed with 0.5% crystal violet solution.

**Wound healing assay**

Confluent monolayers were established by seeding stable gene-modified cells at 3×10⁵ cells per 6-well plate. Mechanical wounding was performed with a 10-μL pipette tip to generate standardized gaps. Following PBS removal of non-adherent cells, serum-restricted conditions were applied, with wound closure monitored at baseline and 24-hour timepoints.

**Transwell migration and invasion assay**

Cell migratory and invasive capacities were assessed using Transwell chambers, either pre-coated with Matrigel (BD Biosciences) or left uncoated. Cellular suspensions were plated in the upper compartment containing serum-deprived medium, while complete growth medium was added to the lower wells. Following 48-hour incubation, after the Transwell chamber culture is completed, discard the culture medium in the wells and gently wash twice with PBS to remove the non-migrated/invaded cells.Fix the cells with 4% paraformaldehyde for 20 minutes, stain them with 0.1% crystal violet solution at room temperature for 15 minutes, and then gently wipe off the cells that did not penetrate the upper membrane surface with a cotton swab.Under an inverted microscope, randomly select 5 fields of view (100× or 200×) and count the number of cells that penetrated the polycarbonate membrane in each field. The experiment is independently repeated 3 times, and the average value is taken as the quantitative index for the migration/invasion ability of each group of cells.

**Animal models**

Female BALB/c nude mice (4-6 weeks old) were obtained from Beijing Vital River Laboratory Animal Technology Co., LTD. Mice were housed under specific pathogen-free conditions and in accordance with animal care institutional guidelines. The mouse model of subcutaneous transplanted tumor was established by subcutaneous injection of 2×10^6^ HCCLM3 cells diluted in 200µL PBS. At 6 weeks, mice were euthanized via cervical dislocation under deep anesthesia induced by intraperitoneal injection of pentobarbital sodium (50 mg/kg). Death was confirmed by the absence of respiratory movement and cardiac pulse. Tumor tissues were then harvested, and tumor volumes were calculated.

**Gene Set Enrichment Analysis**

DEseq2 (1.26.0) R package was used to analyze the differentially expressed genes (degrees) between high and low RBM27 expression samples in TCGA database by Student's t test. An adjusted p value of less than 0.05 and an absolute log2 fold change of more than 1.5 were considered statistically significant. Volcano maps and heat maps were constructed to visualize DEGs.

Pathway enrichment analyses were performed with the “clusterProfiler” R package.GO term analysis, KEGG pathway analysis, and gene set enrichment analysis (GSEA) were performed to elucidate the biological function of RBM27 expression, with an enrichment score | NSE | > 1 (*p* < 0.05), and the five most relevant signal pathways were selected. The c5.all.v7.2. symbols.gmt, and c2.cp.v7.2. symbols.gmt curated gene sets were retrieved from the Molecular Signatures Database (MSigDB). (6-8).

# Supplementary Figures and Tables

## Supplementary Figures


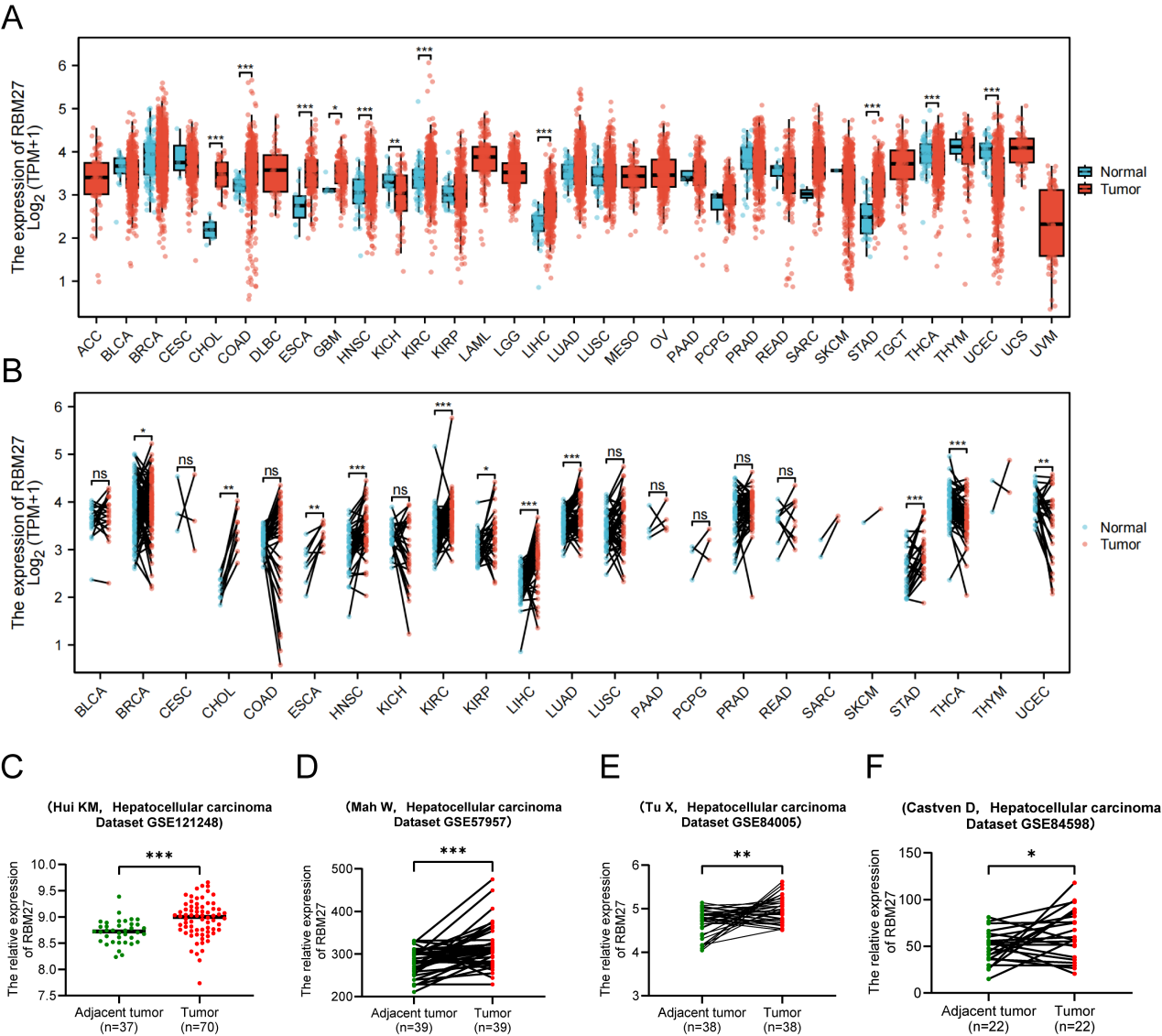
**Supplementary Figure 1 (A)** Comparison of RBM27 expression levels in different cancer tissues and normal tissues.**(B)** The expression levels of RBM27 in different paired cancer tissues and normal tissues were compared.**(C-F)** GEO databases were used to analyze the expression of RBM27 in HCC tissues.

**Supplementary Tables**

**Supplementary Table 1.** Characteristics of patients with HCC in TCGA.

**Supplementary Table 2.** Correlation between clinicopathological variables and RBM27 expression.

**References**

1. Thorsson V, Gibbs DL, Brown SD, Wolf D, Bortone DS, Ou Yang TH, et al. The Immune Landscape of Cancer. Immunity. 2018 Apr 17;48(4):812-830.e14. doi: 10.1016/j.immuni.2018.03.023.

2. Xu L, Deng C, Pang B, Zhang X, Liu W, Liao G, et al. TIP: A Web Server for Resolving Tumor Immunophenotype Profiling. Cancer Res. 2018 Dec 1;78(23):6575-6580. doi: 10.1158/0008-5472.CAN-18-0689.

3. Ritchie ME, Phipson B, Wu D, Hu Y, Law CW, Shi W, et al. limma powers differential expression analyses for RNA-sequencing and microarray studies. Nucleic Acids Res. 2015 Apr 20;43(7):e47. doi: 10.1093/nar/gkv007.

4. Shi J, Wei X, Xun Z, Ding X, Liu Y, Liu L, et al .The Web-Based Portal SpatialTME Integrates Histological Images with Single-Cell and Spatial Transcriptomics to Explore the Tumor Microenvironment. Cancer Res. 2024 Apr 15;84(8):1210-1220. doi: 10.1158/0008-5472.

5. Xun Z, Ding X, Zhang Y, Zhang B, Lai S, Zou D, et al. Reconstruction of the tumor spatial microenvironment along the malignant-boundary-nonmalignant axis. Nat Commun. 2023 Feb 20;14(1):933. doi: 10.1038/s41467-023-36560-7.

6. Love MI, Huber W, Anders S. Moderated estimation of fold change and dispersion for RNA-seq data with DESeq2. Genome Biol. 2014;15(12):550. doi: 10.1186/s13059-014-0550-8.

7. von Mering C, Jensen LJ, Snel B, Hooper SD, Krupp M, Foglierini M, et al. STRING: known and predicted protein-protein associations, integrated and transferred across organisms. Nucleic Acids Res. 2005 Jan 1;33(Database issue):D433-7. doi: 10.1093/nar/gki005.

8. Hänzelmann S, Castelo R, Guinney J. GSVA: gene set variation analysis for microarray and RNA-seq data. BMC Bioinformatics. 2013 Jan 16;14:7. doi: 10.1186/1471-2105-14-7.
